# Unraveling the Roles of Amines in Atom Transfer Radical Polymerization in the Dark

**DOI:** 10.1021/jacs.4c18496

**Published:** 2025-04-02

**Authors:** Arman Moini Jazani, Gorkem Yilmaz, Mitchell Baumer, Julian Sobieski, Stefan Bernhard, Krzysztof Matyjaszewski

**Affiliations:** Department of Chemistry, Carnegie Mellon University, 4400 Fifth Avenue, Pittsburgh, Pennsylvania 15213, United States

## Abstract

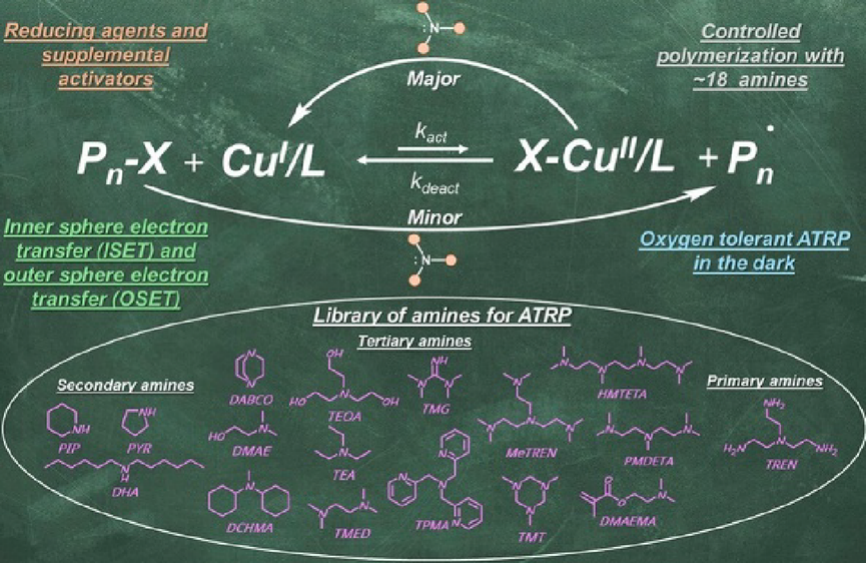

Multidentate amines have been widely used as ligands (L) for Cu-catalysts in atom transfer radical polymerization (ATRP) and as electron donors in photochemically induced polymerizations. However, mechanistic aspects of the role of amines in ATRP in the dark have remained elusive. Herein, the structure–activity relationship and the related electron transfer reactions with Br–Cu^II^/L complexes and/or with alkyl bromides (R-Br) were investigated for 25 amines. Amines function as electron donors and reducing agents for Br–Cu^II^/L complexes via an outer sphere electron transfer (OSET) mechanism, enabling slow but continuous generation of Cu^I^/L activators and inducing controlled ATRP. However, two amines, diazabicyclo(5.4.0)undec-7-ene (DBU) and 1,1,3,3-tetramethylguanidine (TMG), reduced Br–Cu^II^/L faster, suggesting an inner sphere electron transfer (ISET) process. ATRP, starting with initial deactivators (Br–Cu^II^/L) species, proceeded in the dark in the presence of an excess of tertiary amines, such as tris[2-(dimethylamino)ethyl]amine (Me_6_TREN), 1,4-diazabicyclo[2.2.2]octane (DABCO), and TMG at room temperature and afforded polymers with low dispersities (*Đ* ≤ 1.15). With copper(II) triflate complex (Cu^II^/L^+2^, ^–^(OTf)_2_), which has a more positive reduction potential, ATRP proceeded at room temperature with several inexpensive secondary and tertiary amines including triethylamine (TEA) and dimethylethanolamine (DMAE). Interestingly, multidentate amines also served as direct R-Br activators at elevated temperatures (60 °C). In all cases, chains were initiated with R-Br and not by the amine radical cations as byproducts of electron transfer. Amines also enabled ATRP in the presence of residual air in flasks with a large headspace, underpinning them as a robust and accessible reducing agent for practical applications.

## Introduction

Atom Transfer Radical Polymerization (ATRP) is one of the most versatile methods for controlled/living radical polymerization, known as Reversible Deactivation Radical Polymerization (RDRP).^1−6^ This is due to its simplicity and flexibility with a wide range of commercially available monomers, initiators, catalysts/ligands, and undemanding reaction conditions. ATRP operates by reversibly and intermittently activating alkyl halide initiators (R-X) or dormant species (P_n_-X) by Cu^I^/L (L: multidentate amine-based ligand) activators to generate carbon-centered radicals and X-Cu^II^/L deactivators. The ATRP dynamic equilibrium regulates the concentration of radicals (P_n_^•^), diminishes radical termination, and assures concurrent growth of all polymer chains in a controlled/”living” fashion.^7,8^ However, the traditional ATRP method has a drawback – it requires a significant amount of catalyst to compensate for unavoidable radical terminations.^9−11^

**Figure 1:**
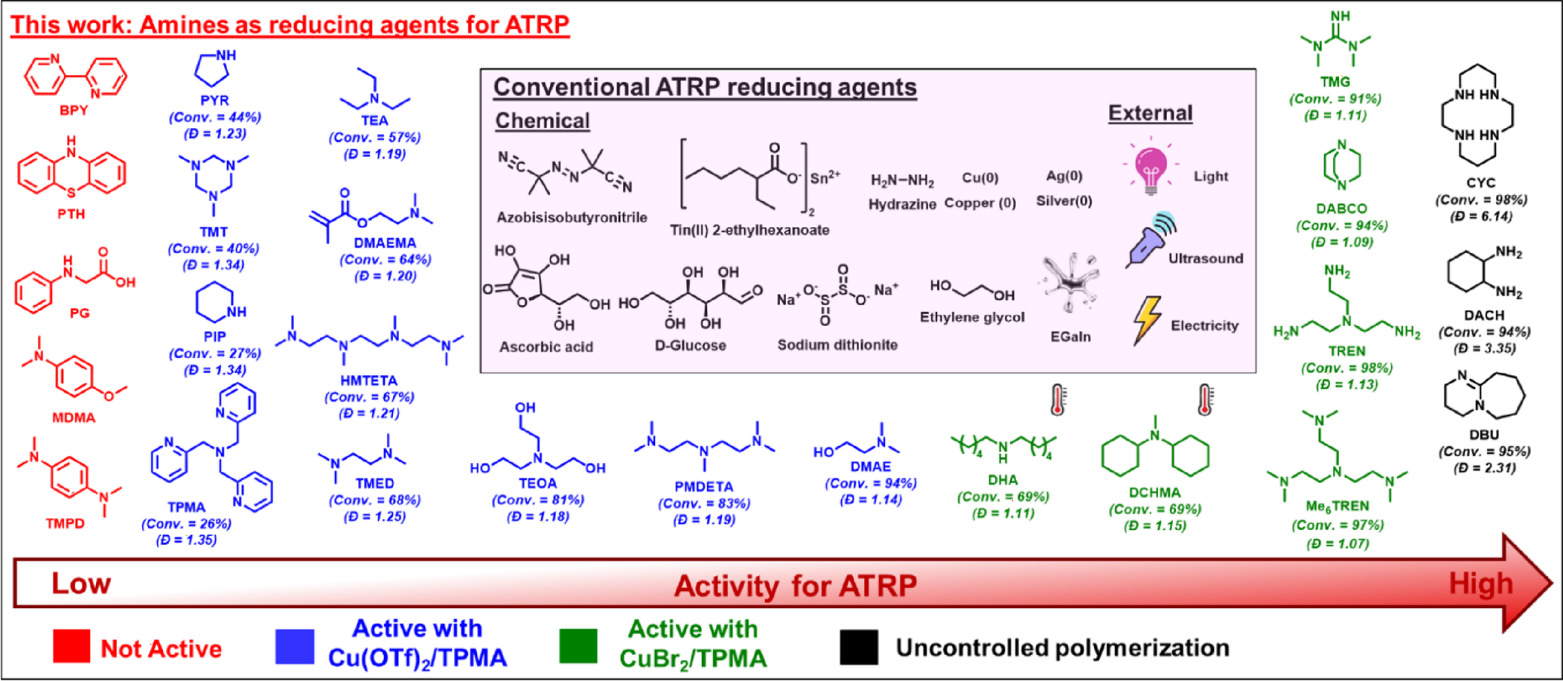
Amines as reducing agent for ATRP compared to conventional chemical and external reducing agents. The arrow represents the effectiveness of amines as reducing agents for ATRP using the CuBr_2_/TPMA or Cu(OTf)_2_/TPMA complex. DBU: 1,8-diazabicyclo(5.4.0)undec-7-ene; DACH: 1,2-diaminocyclohexane; CYC: 1,4,8,11-tetraazacyclotetradecane; Me_6_TREN: tris[2-(dimethylamino)ethyl]amine; TREN: tris(2-aminoethyl)amine; DABCO: 1,4-diazabicyclo[2.2.2]octane; TMG: 1,1,3,3-tetramethylguanidine; DCHMA: *N,N*-dicyclohexylmethylamine; DHA: dihexylamine; DMAE: *N,N*-dimethylaminoethanol; PMDETA: *N,N,N′,N″,N′′*-pentamethyldiethylenetriamine; TEOA: triethanolamine; TMED: tetramethylethylenediamine; HMTETA: 1,1,4,7,10,10-hexamethyltriethylenetetramine; DMAEMA: 2-(dimethylamino)ethyl methacrylate; TEA: triethylamine; PYR: pyrrolidine; TMT: 1,3,5-trimethyl-1,3,5-triazinane; PIP: piperidine; TPMA: tris(2-pyridylmethyl)amine; TMPD: *N,N,N′,N′*-tetramethyl-*p*-phenylenediamine; MDMA: 4-methoxy-*N,N*-dimethylaniline; PG: *N*-phenylglycine; PTH: phenothiazine, BPY: 2,2‘-bipyridyne. The conversion and dispersity values under each amine are according to Table 3 and Table 4.

To address this issue, new efficient techniques have emerged involving the continuous regeneration of Cu^I^/L activators from small amounts (ppm) of oxidatively stable X-Cu^II^/L complexes (deactivators) via various reduction processes. These procedures include activators generated by electron transfer (AGET),^12−14^ activators regenerated by electron transfer (ARGET),^15−18^ initiators for continuous activator regeneration (ICAR),^15,19,20^ and supplementary activator and reducing agent (SARA) ATRP.^21−23^ These approaches employ either chemical reducing agents or external stimuli such as electrochemical, photochemical, and mechanical energy, including ultrasound and tribochemical activation (Figure 1).^24−37^ While the ARGET method employs reducing agents such as Sn(II) 2-ethylhexanoate, silver, hydrazine, or ascorbic acid,^38−43^ SARA primarily utilizes zerovalent Cu^0^, which serves as a supplemental activator and reducing agent in comproportionation (Figure 1). It should be noted that R-X is predominantly (>99%) activated by Cu^I^/L via an inner sphere electron transfer (ISET, or atom transfer) mechanism, which is significantly faster (>billion times) than the outer sphere electron transfer (OSET), as reported in the literature.^40,44,45^

The selection of ligands in ATRP is a critical factor that affects the catalyst solubility and redox potential, thus, the reaction kinetics. Commonly used ligands include substituted 2,2’-bipyrdines (BPY),^46^ tris(2-pyridylmethyl)amine (TPMA),^47^ tris(2-(dimethylaminoethyl)amine (Me_6_TREN),^48^ and *N,N,N’,N”,N”-*pentamethyldiethylenetriamine (PMDETA).^49,50^ These ligands are typically used at a 1:1 ratio with copper halides when tetradentate or tridentate.

Amines play a pivotal role as electron donors, especially in photoATRP systems. When exposed to UV irradiation, photoexcited X-Cu^II^/L is reduced by the amines (e.g., excess ligands) to Cu^I^/L activators.^51−54^ Photoexcitation makes X-Cu^II^/L strongly oxidizing. For instance, the redox potentials of [(Br–Cu^II^/TPMA)^+^]* and (Br–Cu^II^/TPMA)^+^ are +2.07 V and −0.23 V vs SCE, respectively.^55,56^ This substantial difference in reduction potentials should lead to a ca. 10^40^ times faster reduction with amines as electron donors (ca. + 0.7 V vs SCE) after excitation under UV-light irradiation.^55,57^

Amines were also used as reducing agents without light irradiation. For example, 2-(dimethylamino)ethyl methacrylate was polymerized using a CuCl_2_/TPMA catalyst without other external reducing agents.^58^ The tertiary amine groups present in the monomer served as reductants to generate (Cu^I^/TPMA)^+^ Br^–^. Other studies showed that *N,N,N’,N’*-tetramethylethylenediamine (TMEDA), PMDETA, 1,1,4,7,10,10-hexamethyltriethylenetetramine (HMTETA), and Me_6_TREN served dual functions when used in excess amounts, acting as both ligands and reducing agents in ARGET ATRP.^59−62^ Triethylamine (TEA) was also reported as reducing agent for ARGET ATRP at high temperatures (≥80 °C) or as promoters of polymerization rate in conventional ATRP.^63,64^ In a recent study, ARGET of methyl acrylate (MA) with very low amounts of highly active (Cu^I^/Me_6_TREN)^+^Br^–^ rapidly generated radicals which irreversibly terminated (accompanied by a ∼ 5% loss of initiator) and formed in situ Br–Cu^II^/Me_6_TREN.^62,65^ Thus-formed Br–Cu^II^/L^+-^Br deactivator was then slowly reduced by the excess Me_6_TREN present in the medium to regenerate Cu^I^/Me_6_TREN, which activated the remaining alkyl halide initiator.

Although certain ligands used in excess amounts facilitated ARGET ATRP even without light irradiation, there is no comprehensive study on the structure–activity of amines in ATRP systems, their electron transfer pathways, and the mechanistic details of radical generation. In this investigation, we performed experimental, mechanistic, and kinetic studies to understand the role of amines in ATRP without light irradiation. Excess amounts of amines (20-fold excess) with respect to (Br–Cu^II^/L)^+^Br^–^ were used, and their roles in ATRP were studied. Figure 1 illustrates the range of amines studied as both reducing agents and supplemental activators, as will be explained in detail later.

## Results and Discussion

### Thermodynamics and Kinetics of the Reduction of the Deactivators by Amines

The knowledge of redox properties and rate constants of reduction of deactivators (Br–Cu^II^/L^+-^Br) are important for understanding the role of amines in ATRP. Therefore, cyclic voltammetry analysis was first carried out to determine the redox potentials of amines in acetonitrile (MeCN) at room temperature (the oxidation potentials of the amines to their respective amine radical cations were determined and reported as reduction potential, *E*_red,amine_, Table 1 and Supporting Information: Table S1–S4 and Figure S1–S2). Redox potentials and polymerization kinetics were investigated using various amines (structures shown in Figure 1) including primary amines (TREN, 1,2-diaminocyclohexane (DACH)), secondary amines (1,4,8,11-tetraazacyclotetradecane (CYC), dihexylamine (DHA), piperidine (PIP), pyrrolidine (PYR)), tertiary amines (diazabicyclo(5.4.0)undec-7-ene (DBU), 1,1,3,3-tetramethylguanidine (TMG), *N,N*-dicyclohexylmethylamine (DCHMA), tetramethylethylenediamine (TMED), 2-(dimethylamino)ethyl methacrylate (DMAEMA), 1,3,5-trimethyl-1,3,5-triazinane (TMT), triethylamine (TEA), triethanolamine (TEOA), *N,N*-dimethylaminoethanol (DMAE) and 1,4-diazabicyclo[2.2.2]octane (DABCO)), aryl amines (*N*-phenylglycine (PG), *N,N,N′,N′*-tetramethyl-p-phenylenediamine (TMPD), 4-methoxy-*N,N*-dimethylaniline (MDMA), phenothiazine (PTH)), aromatic amines (BPY), and four ATRP ligands (TPMA, Me_6_TREN, HMTETA, and PMDETA).

**Table 1:** Redox Potentials of Various Amines Used for ATRP^a^

Entry	Amine	*E*_red,amine_^a^ (V vs SCE) (1.0 V/s)
**Tertiary Amines**		
1	TMG	1.25
2	TMT	1.05
3	DBU	1.04
4	DMAEMA	1.00
5	TPMA	0.98
6	TMED	0.83
7	TEOA	0.81
8	DMAE	0.75
9	TEA	0.69
10	DABCO^b^	0.64
11	HMTETA	0.58
12	PMDETA	0.56
13	DCHMA	0.56
14	Me_6_TREN	0.54
**Secondary Amines**		
15	PIP	1.16
16	DHA	0.98
17	PYR	0.77
18	CYC^c^	-
**Primary Amines**		
19	DACH	1.27
20	TREN^c^	-
**Heteroaromatic/Aryl Amines**		
21	BPY	2.04
22	PG	0.92
23	PTH^b^	0.66
24	MDMA^b^	0.60
25	TMPD^b^	0.19

a Redox potential (inflection point) calculated with respect to the saturated calomel electrode (SCE) in MeCN; chemical structures of amines are shown in Figure 1.

b Showed reversible process so only the half-wave potential of the reversible peak was reported.

c TREN and CYC were insoluble in MeCN. The oxidation potentials of the amines to their respective amine radical cations were determined and reported as reduction potentials, *E*_red,amine_.

Amines except for PTH, MDMA, TMPD, and DABCO displayed irreversible oxidation. Therefore, the inflection point values were used for calculations except for reversible amines where half-wave potentials were used, as reported in literature.^66^ The redox potential of amines did not significantly change with different scan rates (0.1, 0.5, 1 V/s) (Figure S1).^66^ In general, ligand-like tertiary amines (Me_6_TREN, PMDETA and HMTETA) showed lower *E*_red,amine_ values than other tertiary amines.

The reversible half-wave redox potentials of (Br–Cu^II^/ TPMA)^+^ Br^–^ and (Br–Cu^II^/ Me_6_TREN)^+^ Br^–^ were determined as *E*_red Cu/L_ = −0.242 V and −0.330 V, respectively.^56,67^ These reduction potential values were used to calculate the free energy (Δ*G*) (eq 1 for an outer sphere electron transfer (OSET) process (eq 2. Then, the (pseudo)equilibrium constants (*K*_eq_) of each redox reaction were estimated (eq 3:
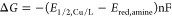
1
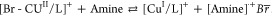
2
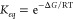
3

The Δ*G* values for the forward reaction are highly positive (*K*_eq_ ≪ 1), suggesting the reduction of (Br–Cu^II^/L)^+^ Br^–^ should occur very slowly in the dark. The reduction of amine radical cations to their neutral state (backward reaction) was assumed to be diffusion-controlled (*k*_ox_ ∼ 10^10^ M^–1^s^–1^).^68,69^ This value was used to estimate the reduction rate constants via OSET (*k*_red(theo)_), according to eq 4:
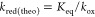
4

The experimental reduction kinetics (Table 2, *k*_red(exp)_) of (Br–Cu^II^/TPMA)^+^ Br^–^ by excess amines and (Br–Cu^II^/Me_6_TREN)^+^ Br^–^ by excess Me_6_TREN was determined by following the changes in the respective absorption (A) at 960 and 970 nm using a UV–vis-NIR spectrophotometer (Figure S3). The rate constants of reductions were calculated using pseudo-first-order kinetics as shown in eq 5.
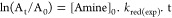
5

**Table 2:** Redox Potentials of Amines and Kinetics and Thermodynamic Values of Their Electron Transfer with (Br–Cu^II^/TPMA)^+^ Br^–^a

Entry	Amine	*E*_red,amine_^a^ (V vs SCE)	Δ*G* (kJ·mol^–1^)	*K*_eq_ (*k*_red_/*k*_ox_)	*k*_red(theo)_^b^ (M^–1^s^–1^)	*k*_red(exp)_^c^ (M^–1^s^–1^)
1	PMDETA	0.56	77.4	2.7 × 10^–14^	2.7 × 10^–4^	2.9 × 10^–5^
2	HMTETA	0.58	79.3	1.2 × 10^–14^	1.2 × 10^–4^	2.1 × 10^–5^
3	Me_6_TREN^d^	0.54	83.9	1.9 × 10^–15^	1.9 × 10^–5^	1.8 × 10^–5^
4	DABCO	0.64	85.1	1.2 × 10^–15^	1.2 × 10^–5^	1.1 × 10^–5^
5	TPMA	0.98	117.9	2.1 × 10^–21^	2.1 × 10^–11^	-e
6	DBU	1.04	123.7	2.1 × 10^–22^	2.1 × 10^–12^	5.0 × 10^–2^
7	TMG	1.25	143.9	5.8 × 10^–26^	5.8 × 10^–16^	1.2 × 10^–2^

a The redox peak values (inflection point) calculated with respect to the saturated calomel electrode (SCE) in MeCN.

b All calculations were based on 1.0 V/s scan rate and calculated based on *K*_eq_, estimating that the back reaction’s rate: *k*_ox_ = 10^10^ M^–1^s^–1^ and half-wave redox potential of (Br–Cu^II^/ TPMA)^+^ Br^–^: *E*_1/2_ = −0.242 V vs SCE.

c Calculated based on UV–vis-NIR analysis (Figure S3).

d In the case of Me_6_TREN, no TPMA was used. Redox potential of (Br–Cu^II^/ Me_6_TREN)^+^ Br^–^: *E*_1/2_ = −0.330 V vs SCE was used for calculations.

e No reduction was observed.

The reduction rate constants (*k*_red(theo)_) calculated for ligand-like amines (entries 1–3, Table 2) and DABCO are in approximate agreement with those measured by UV analyses (*k*_red(exp)_), confirming an OSET mechanism (Table 2). The small difference between *k*_red(theo)_ and *k*_red(exp)_ might be due to different diffusion coefficients of amines, solvent viscosity, and selection of a peak redox potential rather than the inflection point values. However, for TMG and DBU, *k*_red(exp)_ values were significantly higher than theoretically predicted values (Table 2, entries 6 and 7). The reduction of CuBr_2_/TPMA by DBU was so rapid that almost all Cu(II) species were reduced to Cu(I) before the first UV–vis spectrum was measured (Figure S3).

In contrast to the OSET process, where the primary coordination spheres remain intact, ISET (inner sphere electron transfer or atom transfer) proceeds by a bridged concerted pathway and can be very strongly accelerated. A classic example of successful ISET is the activation step in ATRP, where the halogen atom is transferred from the alkyl halide to Cu^I^/L, generating an alkyl radical and X-Cu^II^/L deactivator. ISET proceeds via a bent transition state and is much faster (10^11^ times) than the theoretical values for OSET predicted based on Marcus’ theory.^45,70^ Thus, the substantially faster reduction with TMG and DBU can be attributed to the ISET mechanism. Nevertheless, the contribution of other pathways, such as acceleration of reduction by coordinated bases or deprotonated solvent, cannot be fully ruled out.

The electron transfer process in the presence of the multidentate amines was studied at 25, 40, and 60 °C. The reactions were much faster at higher temperatures (Figure S4). By plotting the logarithmic reduction rate constant values vs inverse absolute temperature, 1/K, (eq 6, the activation energies (*E*_a_) of the OSET processes were calculated as 80, 92, and 90 kJ/mol for PMDETA, Me_6_TREN, and HMTETA, respectively (Figure S 4d). These values could be compared to activation energies of ca. 30 kJ/mol for various alkyl halides by CuBr/PMDETA and 130 kJ/mol for AIBN decomposition.^71,72^ These values clearly indicate strong acceleration of the reduction of Br–Cu^II^/TPMA by amines at higher temperatures.
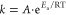
6

### Investigation of the Effect of Various Amines on ATRP in the Presence of CuBr_2_

The ATRP in the presence of various amines as reducing agents was investigated in the dark (without exposure to room light). To conduct polymerization experiments, copper complexes with appropriate redox properties, sufficient activity in their lower oxidation state, and stability in the presence of excess amounts of amines to avoid ligand-exchange reactions were selected. The relative binding strengths of TPMA, Me_6_TREN, PMDETA, and HMTETA ligands with CuBr_2_ were tested by UV–vis-NIR spectroscopy. The absorption spectra of each complex were monitored after the addition of an external amine to compare their relative binding strength (stability) with CuBr_2_. The stabilities of CuBr_2_ complexes follow the order: Me_6_TREN > TPMA > HMTETA > PMDETA (Figure S5). Similar analyses in water revealed a different order: TPMA > Me_6_TREN > HMTETA > PMDETA. This order agrees with data reported in the literature.^73,74^ Based on these results, TPMA was used as the standard ligand as it exhibits mild reduction potential (see *infra*) and could not be replaced by most of the amines except Me_6_TREN in organic solvents.

Methyl acrylate (MA) was polymerized under a nitrogen blanket using ethyl 2-bromoisobutyrate (EBiB) as the initiator, (Br–Cu^II^/L)^+^ Br^–^ as the initial Cu complex deactivator, and various amines used as reducing agents in a 20-fold excess. The following molar ratios were used: [MA]_0_/[EBiB]_0_/[CuBr_2_]_0_/[TPMA]_0_/[amines]_0_ = 100/1/0.02/0.02/0.4, [MA]_0_ = 5.8 M in DMSO. The polymerization solutions were kept in the dark to prevent a possible impact of light sensitization.

Table 3 presents the results of the polymerizations (cf. also Figure 1 and Figure S6 for GPC traces). Ligand-like tertiary amines (e.g., Me_6_TREN) promoted the ARGET ATRP process at room and higher temperature (23 or 60 °C), allowing control over molecular weights and dispersity (*Đ* ≤ 1.09). Nonligand tertiary amines such as DABCO and TMG led to controlled polymerization with a lower initiation efficiency, which could be related to their higher basicity and higher nucleophilicity.^75^ Polymerization mediated by DBU was uncontrolled with a viscous reaction mixture formed after 5 h. A loss of control in the system could be attributed to the too fast reduction and the loss of the deactivator. Also, possible side reactions, such as the elimination of the halide chain ends from the dormant species in the presence of very basic amines, could affect the results (Figure S7). No polymerization was observed with other tertiary amines: DMAE, PMDETA, TEOA, TEA, TMED, HMTETA, DMAEMA, TMT, and TPMA, even at 60 °C (Table S5). Interestingly, ligands (i.e., TPMA, PMDETA and HMTETA) with lower purity (yellowish, used as received or not properly stored) were sometimes active with (Br–Cu^II^/TPMA)^+^ Br^–^ (Table S6), underscoring the importance of using purified ligands to obtain reproducible and consistent ATRP kinetic results.

**Table 3:**
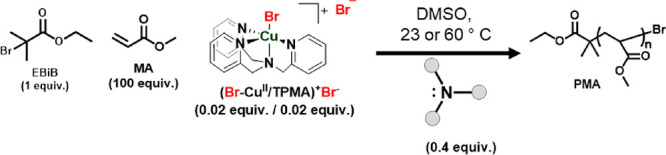
ATRP of MA Using (Br–Cu^II^/TPMA)^+^ Br^–^ Complex and Various Amines^a^

Entry	Amines	Time (h)	Temp (°C)	Conv.^b^ (%)	*M*_n,theo_^c^	*M*_n,GPC_^d^	*Đ*^d^	I* (%)^e^
1^f^	Me_6_TREN	24	23	97	8500	9800	1.07	87
2^f^	Me_6_TREN	5.5	60	91	8000	8450	1.09	95
3	DABCO	24	23	94	8300	13200	1.09	63
4	TMG	24	23	91	8000	10700	1.11	74
5	DBU	24	23	95	8200	32200	2.31	26
6	DCHMA	24	23	14	1400	1100	1.35	127
7	DCHMA	5.5	60	69	6100	5800	1.15	106
8	DHA	5.5	60	69	6100	5800	1.11	105
9	CYC	24	23	98	8600	34500	6.14	25
10	TREN	24	23	98	8600	9400	1.13	92
11	DACH	24	23	94	8300	14000	3.35	59

a Reaction conditions: [MA]_0_/[EBiB]_0_/[CuBr_2_]_0_/[TPMA]_0_/[amines]_0_ = 100/1/0.02/0.02/0.4 in DMSO, [MA]_0_ = 5.8 M.

b Calculated by ^1^H NMR.

c Calculated using the formula: *M*_n,theo_ = Conv(%) × MW_MA_ × DP + MW_EBiB_.

d Determined by GPC using PMMA standards.

e Calculated using the equation: Initiation efficiency (I* (%)) = *M*_n,theo_/*M*_n,GPC_ × 100.

f No TPMA was used in the case of Me_6_TREN. No polymerization was observed with TEA, TEOA, TMED, DMAE, DMAEMA, TMT, PMDETA, HMTETA, TPMA, PYR, PIP, PG, PTH, TMPD, MDMA, and BPY.

Increasing the temperature to 60 °C provided almost quantitative conversions at shorter reaction times (5.5 h) for secondary and tertiary amines, corroborating the faster reduction of Br–Cu^II^/L at elevated temperatures. A very slow polymerization with DCHMA at room temperature was significantly accelerated at 60 °C with preserved control. Polymerizations mediated by secondary amines, such as dihexylamine (DHA), proceeded at a slower rate at room temperature (14% after 24 h), but were accelerated at 60 °C. No polymerization was observed with secondary cyclic amines (i.e., PIP and PYR). Initiation efficiency (I*) values remained high, indicating low contribution of the plausible chain transfer reactions.^76^ Polymerization in the presence of ligand-like secondary amines such as CYC was fast and uncontrolled, which could be ascribed to the competitive complexation with the Br–Cu^II^/TPMA^+^ Br^–^ complex.

Except for TREN, which can also operate as a ligand, uncontrolled polymerizations were observed when primary amines were used (i.e., DACH). This suggests the possibility of additional initiation pathways or degradative side reactions (e.g., Michael addition, displacement of terminal halogen). Finally, polymerization was unsuccessful in the presence of all tested aryl amines (i.e., PG, TMPD, MDMA, PTH) and aromatic amines (i.e., BPY), even after 72 h or at higher temperatures (60 °C for 5.5 h). Aryl and aromatic amines have no α-hydrogen to form the downstream iminium ions; therefore, they could participate in back-electron-transfer (BET) to yield (Cu^II^/TPMA)^+^ Br^–^ and neutral amines. This was confirmed by CV analysis, which revealed a reversible cyclic voltammogram for PTH, MDMA, and TMPD (Figure S2 and Table S4).^77,78^

### ATRP via Reduction of Copper(II) Triflate with Amines

Since ARGET was the predominant mechanism for ATRP with amines facilitated by the reduction of Br–Cu^II^/L, it should be even more efficient with a faster reduction with (Cu^II^/TPMA)^2+-^(OTf)_2_ which has more positive redox potential (*E*_1/2_ = −0.022 V vs SCE) than (Br–Cu^II^/TPMA)^+^ Br^–^ (*E*_1/2_ = −0.242 V vs SCE) (Figure S8). Therefore, (Cu^II^/TPMA)^2+-^(OTf)_2_ was used as the catalyst for ATRP.^79^ Excitingly, several very inexpensive and accessible amines (i.e., TEA, TEOA, DMAE, and TMED) that were completely inactive with (Br–Cu^II^/TPMA)^+^ Br^–^ promoted ATRP in the dark when (Cu^II^/TPMA)^2+-^(OTf)_2_ was used (Table 4). Except for arylamines (i.e., PTH, PG, MDMA, TMPD) and the heterocyclic aromatic amine (i.e., BPY) that remained inert for ATRP (Table S7), polymerization was observed with all other amines at room temperature. Notably, all polymerizations were well-controlled (low *Đ* and high I*), and GPC traces were monomodal (Figure S9). It must be stressed that the deactivator, in this case (Br–Cu^II^/TPMA)^+-^OTf was spontaneously generated by the reaction of the dormant species (P_n_-Br) with the reduction product, i.e. activator (Cu^I^/TPMA)^+-^OTf. Slightly higher dispersities in Table 4 are related to a lower concentration of the actual deactivator (Br–Cu^II^/TPMA)^+-^OTf, especially at early point of polymerization. The faster reduction of (Cu^II^/TPMA)^+2–^(OTf)_2_ compared to (Br–Cu^II^/TPMA)^+^ Br^–^ was further confirmed by UV–vis-NIR. Model amines (i.e., TEA, PMDETA, Me_6_TREN and DABCO) reduced (Cu^II^/TPMA)^+2–^(OTf)_2_ faster than (Br–Cu^II^/TPMA)^+^ Br^–^ (Figure S10).

**Table 4:**
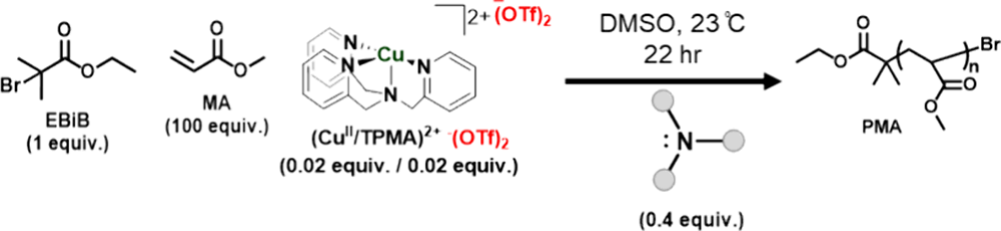
ATRP of MA Using (Cu^II^/TPMA)^2+-^(OTf)_2_ Complex and Various Amines^a^

Entry	Amines	Conv. (%)^b^	*M*_n,theo_^c^	*M*_n,GPC_^d^	*Đ*^d^	I*(%)^e^
1	DMAE	94	8300	7500	1.14	110
2	PMDETA	83	7300	7300	1.19	100
3	TEOA	81	7200	6500	1.18	110
4	DHA	70	6200	6500	1.14	96
5	TMED	68	6000	6100	1.25	99
6	HMTETA	67	6000	6400	1.21	93
7	DMAEMA	64	5700	5200	1.20	109
8	TEA	57	5100	4600	1.19	110
9	PYR	44	4000	3500	1.23	115
10	TMT	40	3600	3700	1.34	98
11	PIP	27	2500	2300	1.34	109
12	TPMA	26	2400	2400	1.35	100

a Reaction conditions: [MA]_0_/[EBiB]_0_/[Cu(OTf)_2_]_0_/[TPMA]_0_/[amines]_0_ = 100/1/0.02/0.02/0.4 in DMSO, [MA]_0_ = 5.8 M. Polymerization was carried out at 23 °C for 22 h.

b Calculated by ^1^H NMR.

c Calculated using the formula: *M*_n,theo_ = Conv(%) × MW_MA_ × DP + MW_EBiB_.

d Determined by GPC using PMMA standards.

e Calculated using the equation: Initiation efficiency (I* (%)) = *M*_n,theo_/*M*_n,GPC_ × 100.

### Oxygen Tolerant ATRP with Amines

Oxygen-tolerant ATRP, in the presence of chemical, photochemical, and biological reagents, has received significant attention in recent years.^6,80,81^ Chemical deoxygenation in ATRP was previously achieved by using a large excess of reducing agents or high temperatures at which oxygen has lower solubility.^82−85^ These approaches enabled ATRP in the presence of a limited amount of oxygen in closed-cap vials with no headspace.^83,86^ By evaluating the efficiency of different amines in ATRP performed under ambient conditions (in the presence of air), we observed that ATRP in the presence of amines proceeded without O_2_ degassing. ATRP with Me_6_TREN and TREN (entries 1 and 3, Table 5) reached high conversions with dispersity values similar to those under deoxygenated conditions (*Đ* ≤ 1.15) with monomodal GPC traces (Figure S11). The agreement between *M*_n,theo_ and *M*_n,GPC_ was slightly compromised in the presence of O_2_, which was attributed to the loss of some chains/radicals by O_2_, yielding unreactive peroxide species.^87,88^ However, some amines that were active under an inert atmosphere did not afford any polymer (i.e., DABCO and DHA) or led to poor polymerization control (i.e., TMG) in the presence of O_2_. Remarkably, oxygen-tolerant ATRP in the presence of (Cu^II^/TPMA)^2+-^(OTf)_2_ proceeded smoothly even in the presence of common inexpensive amines (i.e., TEA, TEOA, and DMAE). All polymerizations were carried out in a vial with 1 mL headspace at room temperature without any light irradiation, demonstrating the robustness of the strategy. Polymerizations in a vial with larger headspace (entries 10–11, Table 5) and even in an open-cap vial (entry 12, Table 5) progressed slower, but without compromising control. The rate of oxygen-tolerant ATRP with amines was significantly enhanced, and initiation efficiency improved at 60 °C (entries 13–14, Table 5). This was attributed to the faster reduction of (Br–Cu^II^/TPMA^+-^Br) with amines and the lower solubility of O_2_ in DMSO at higher temperatures. Oxygen tolerance rendered by amines was attributed to the reaction of amine radical cations/α-aminoalkyl radicals with oxygen and the formation of amides, *N*-formamides, or *N*-oxide species, as reported in the literature (Figure S12).^89−91^ Polymerizations in the presence of oxygen was accompanied by a yellow coloring of the reaction mixture (Figure S13), suggesting the formation of oxidized amine products.

**Table 5:**
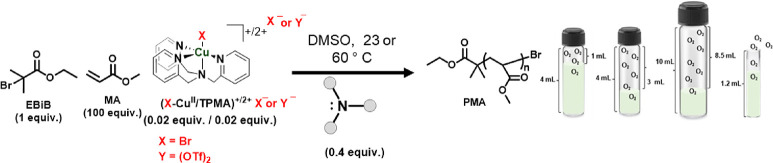
Oxygen Tolerant ATRP of MA Using (Br–Cu^II^/TPMA)^+^ Br^–^ or (Cu^II^/TPMA)^2+-^(OTf)_2_ and Various Amines^a^

Entry	Cu(X)_2_	Amines	Time (h)	Headspace (mL)	Temp (°C)	Conv. (%)^b^	*M*_n,theo_^c^	*M*_n,GPC_^d^	*Đ*^d^	I* (%)^e^
1	CuBr_2_	Me_6_TREN	24	1	23	93	8200	10300	1.09	79
2	CuBr_2_	DCHMA	72	1	23	74	6600	7900	1.16	83
3	CuBr_2_	TREN	24	1	23	67	6000	9600	1.15	62
4	CuBr_2_	TMG	24	1	23	56	5000	6700	1.59	75
5	CuBr_2_	DHA	72	1	23	<5	-	-	-	-
6	CuBr_2_	DABCO	24	1	23	<5	-	-	-	-
7	Cu(OTf)_2_	DMAE	24	1	23	78	6900	7100	1.17	98
8	Cu(OTf)_2_	TEA	24	1	23	71	6300	6900	1.16	91
9	Cu(OTf)_2_	TEOA	24	1	23	46	4200	4500	1.32	95
10	Cu(OTf)_2_	TEA	30	3	23	62	5500	6000	1.27	92
11	Cu(OTf)_2_	TEA	30	8.5	23	53	4800	5300	1.28	90
12	Cu(OTf)_2_	TEA	42	Open-air	23	46	4200	4000	1.24	103
13	Cu(OTf)_2_	TEA	5.5	3	60	75	6600	6700	1.26	98
14	Cu(OTf)_2_	DMAE	5.5	3	60	73	6500	5600	1.27	116

a Reaction conditions: [MA]_0_/[EBiB]_0_/[CuBr_2_ or Cu(OTf)_2_]_0_/[TPMA]_0_/[amines]_0_ = 100/1/0.02/0.02/0.4 in DMSO, [MA]_0_ = 5.8 M. All polymerizations were carried out in DMSO

b Calculated by ^1^H NMR.

c Calculated using the formula: *M*_n,theo_ = Conv(%) × MW_MA_ × DP + MW_EBiB_.

d Determined by GPC using PMMA standards.

e Calculated using the equation: Initiation efficiency (I* (%)) = *M*_n,theo_/*M*_n,GPC_.

### Kinetics and Versatility of ATRP with Amines

**Figure 2:**
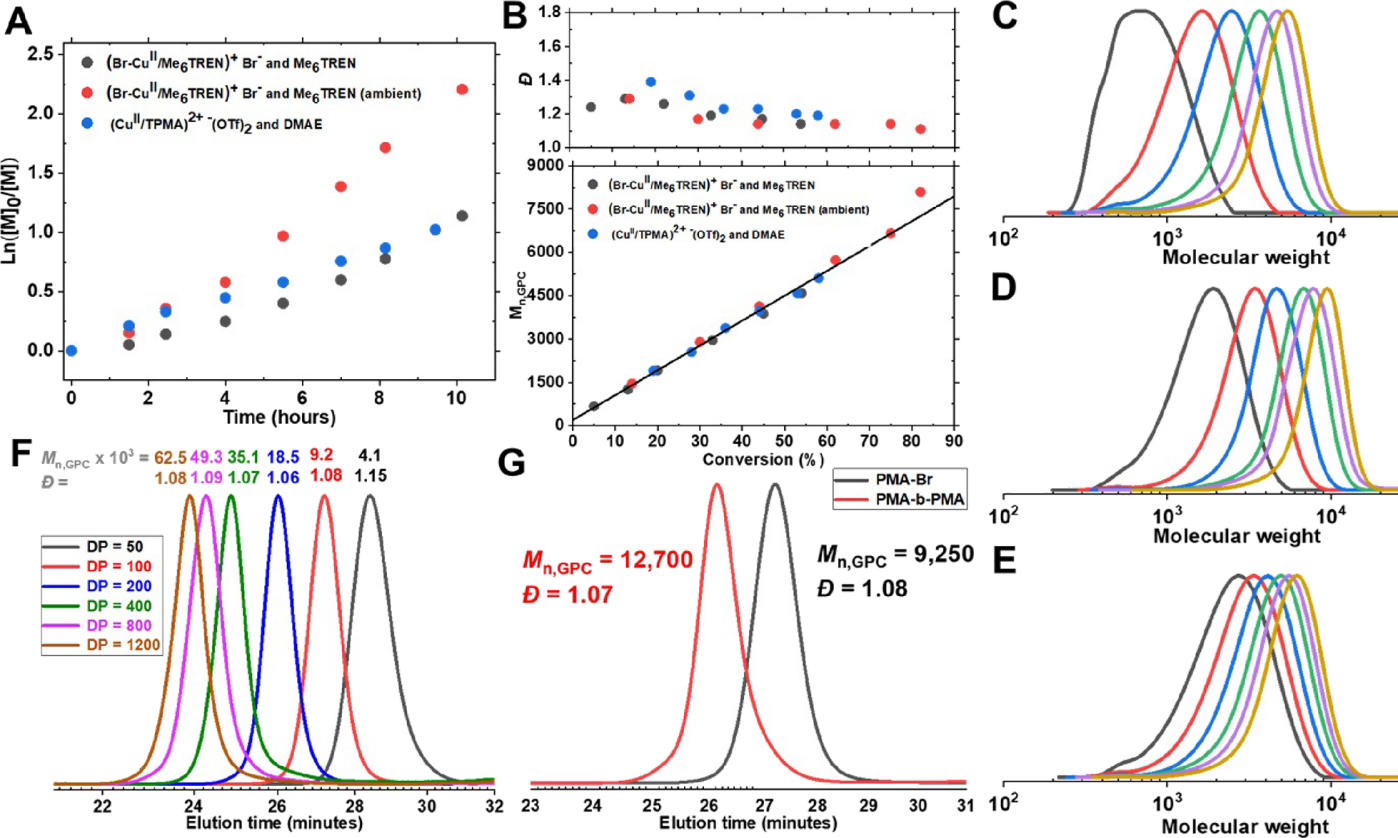
(A) Kinetics of ATRP of MA with amines. (B) Evolution of *Đ* and *M*_n,GPC_ with conversion. Evolution of GPC traces for polymerization with (C) (Br–Cu^II^/Me_6_TREN)^+^Br^–^ and Me_6_TREN (with deoxygenation), (D) (Br–Cu^II^/Me_6_TREN)^+^Br^–^ and Me_6_TREN (ambient), and (E) (Cu^II^/TPMA)^2+-^(OTf)_2_ and DMAE (with deoxygenation). GPC traces of (F) poly(methyl acrylate) synthesized at different target DPs (50–1200) with (Br–Cu^II^/Me_6_TREN)^+^Br^–^ and Me_6_TREN (with deoxygenation), (G) PMA precursor before and after chain extension with (Br–Cu^II^/Me_6_TREN)^+^Br^–^ and Me_6_TREN (with deoxygenation). Reaction conditions: [MA]_0_/[EBiB]_0_/[CuBr_2_ or Cu(OTf)_2_]_0_/[Me_6_TREN or TPMA]_0_/[amines]_0_ = 100/1/0.02/0.02/0.4 in DMSO at 23 °C, [MA]_0_ = 5.8 M.

The kinetics of ATRP of MA in the presence of (Br–Cu^II^/Me_6_TREN)^+^Br^–^ and Me_6_TREN were investigated using model amines under three different conditions with deoxygenation and without deoxygenation (entry 1, Table 3 and entry 1, Table 5) and also using (Cu^II^/TPMA)^2+-^(OTf)_2_ and DMAE as reducing agent (entry 1, Table 4). Semilogarithmic kinetic plots showed mild acceleration with a faster initial stage for (Cu^II^/TPMA)^2+-^(OTf)_2_/DMAE (Figure 2A). The molecular weight distributions of the obtained polymers were relatively narrow (*Đ* < 1.39) and decreased as conversions increased. The experimental values of *M*_n,GPC_ were in agreement with calculated *M*_n,theo_ values (Figure 2B), and the monomodal GPC traces evolved with time (Figure 2C-E). Polymerization with Me_6_TREN was slightly faster in the presence of oxygen than under the deoxygenated conditions, probably due to the formation of a copper superoxido complex, as previously reported in literature.^65,92^ There was no induction period in the ATRP with (Cu^II^/TPMA)^2+-^(OTf)_2_ and DMAE, underpinning the previous hypothesis for faster reduction of (Cu^II^/TPMA)^2+-^(OTf)_2_ and no actual deactivator (Br–Cu^II^/L) which could result in the inhibition period. ATRP with (Br–Cu^II^/Me_6_TREN)^+^Br^–^ was examined for the synthesis of PMA with different targeted degrees of polymerization (DP) (Figure 2F and Table S9). All polymerizations reached high conversions and polymers had narrow molecular weight distribution (1.06 ≤ *Đ* ≤ 1.15) up to DP = 1200 (Figure 2F). Chain extension experiments were performed to test the chain end fidelity of PMA-Br prepared by ATRP with (Br–Cu^II^/Me_6_TREN)^+^Br^–^. Chain extensions with the same catalytic system showed a clear shift in GPC traces to a higher molecular weight range, confirming the high chain-end fidelity of the precursor polymer and the versatility of amines to be used for synthesizing block copolymers (Figure 2G).

### Alkyl Halide Activation by Amines (Supplemental Activation, SA)

**Figure 3:**
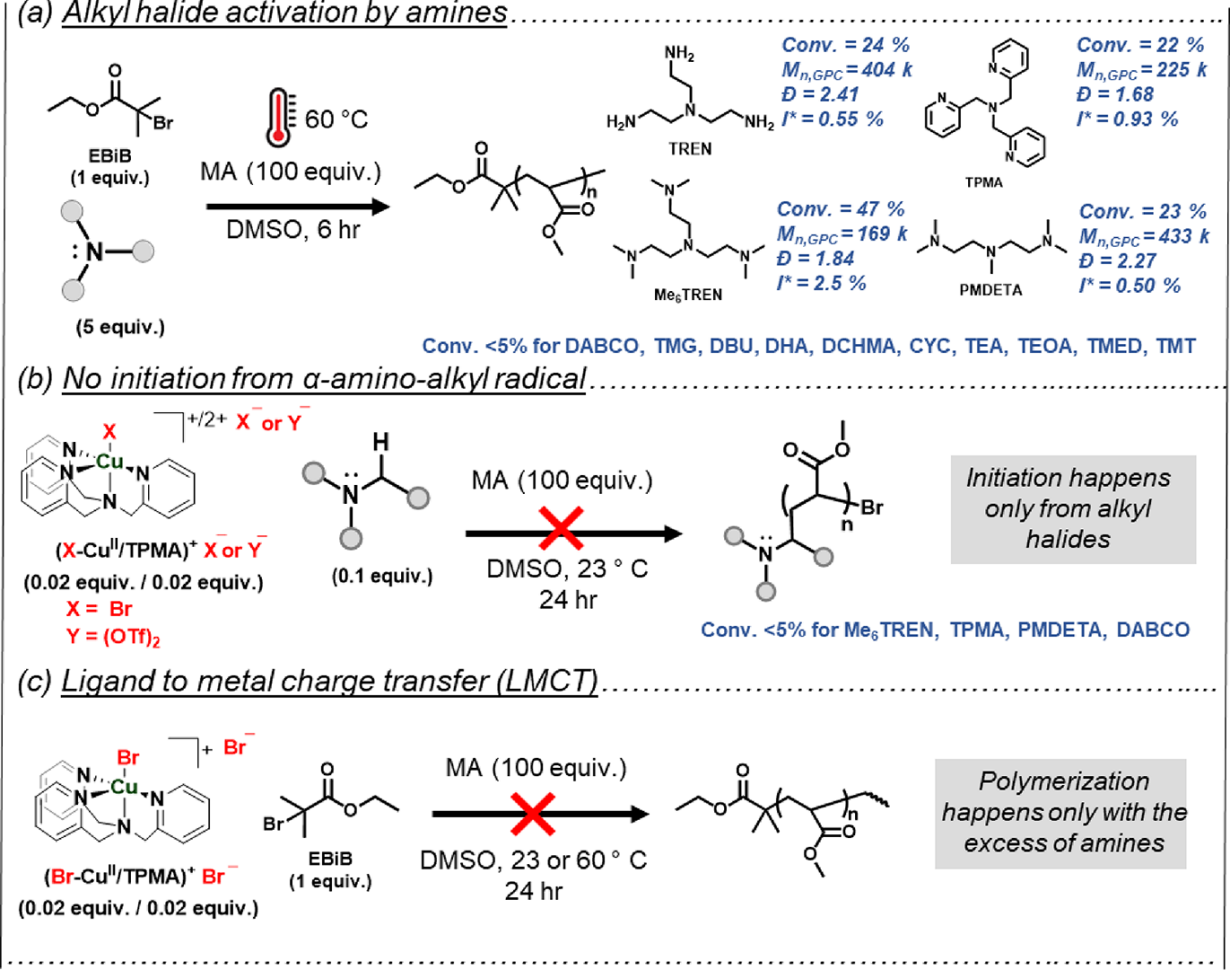
Mechanistic analysis of ATRP with amines by evaluating alternative reaction pathways. (a) supplemental activation of R-Br with amines at 60 °C, (b) no initiation of polymerization from amines without R-Br, (c) no polymerization was observed with equimolar amines.

To better understand the role of amines and their effect on ATRP, the amines were tested as supplemental activators in reactions without (Br–Cu^II^/L)^+^ Br^–^. Relatively slow and uncontrolled polymerizations were observed when the temperature was raised to 60 °C, with only EBiB and ligand-like amines (e.g., Me_6_TREN, TREN, PMDETA, and TPMA), as shown in Figure 3a. Very low initiation efficiencies (<1% for TREN, TPMA, and PMDETA and 2.5% for Me_6_TREN) and polymers with high dispersity values (1.68 ≤ Đ ≤ 2.41) were obtained. This suggested radical generation through EBiB/amine interactions but no deactivation. The radical generation through the homolytic cleavage of R-Br in the presence of amines can be related to the formation of charge transfer complexes [R-Br···Amine] or by halogen bonding, which is more pronounced at elevated temperatures and even more for alkyl iodides.^93−97^ No supplemental activation was observed with alkyl chlorides (Figure S14). Therefore, such amines could display a dual role, acting as both supplemental activators and reducing agents (SARA mechanism). No contribution of SA was observed for simple monodentate amines, even at 60 °C (Table S10). Thus, the geometry/binding of amines is essential for the magnitude of [R-Br···Amine] bonding and the subsequent homolytic dissociation. Accelerated supplemental activation of alkyl bromide by ligand-like amines could be related to a more efficient complexation of alkyl bromides by multidentate amines, with increased halogen bond acceptor capabilities.^98^

The contribution of supplemental activation was analyzed by kinetic measurements at 60 °C. The kinetics of the polymerizations with and without CuBr_2_ were investigated ([MA]_0_/[EBiB]_0_/[CuBr_2_]_0_/[Me_6_TREN]_0_ = 100/1/x/0.1, x: 0.02 or 0). Linear semilogarithmic plots demonstrated controlled polymerization in the presence of CuBr_2_, in which the alkyl halide could be activated both by the (CuI/L)^+^ Br^–^ generated via the reduction with excess ligand and by the supplemental activation (SA) (Figure S15). The *k*_app_ value in the presence of CuBr_2_ was *k*_Cu_ = 1.1 × 10^–4^ M^–1^s^–1^. Without CuBr_2_, conventional radical polymerization was observed, promoted only by SA (Figure S15). The *k*_app_ value of the uncontrolled radical polymerization at the same reaction time (∼ 4 h) was *k*_SA_ = 5.4 × 10^–6^ M^–1^s^–1^. A comparison of the apparent rate constants (eq 7 gave the 4.6% contribution of radical formation through SA. This value is close to the initiation efficiency value (I* = 2.5%, Figure 3a) observed for polymerizations performed with Me_6_TREN in the absence of CuBr_2_.
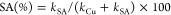
7

Therefore, the predominant pathway affecting the rate of ATRP is the ARGET mechanism (RA), where (Br–Cu^II^/Me_6_TREN)^+^ Br^–^ is reduced by the excess Me_6_TREN, rather than SA.

The semilogarithmic kinetic slopes gave the apparent rate constant (*k*_app_), which is a product of the actual radical propagation rate constant (*k*_p_(MA) = 3 × 10^4^ M^–1^s^–1^ at 60 °C)^99^ and the radical concentration ([R^•^]) (eq 8.
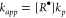
8

The radical concentrations were ca. 4 × 10^–9^ M and 2 × 10^–10^ M with and without Cu catalyst.

**Figure 4:**
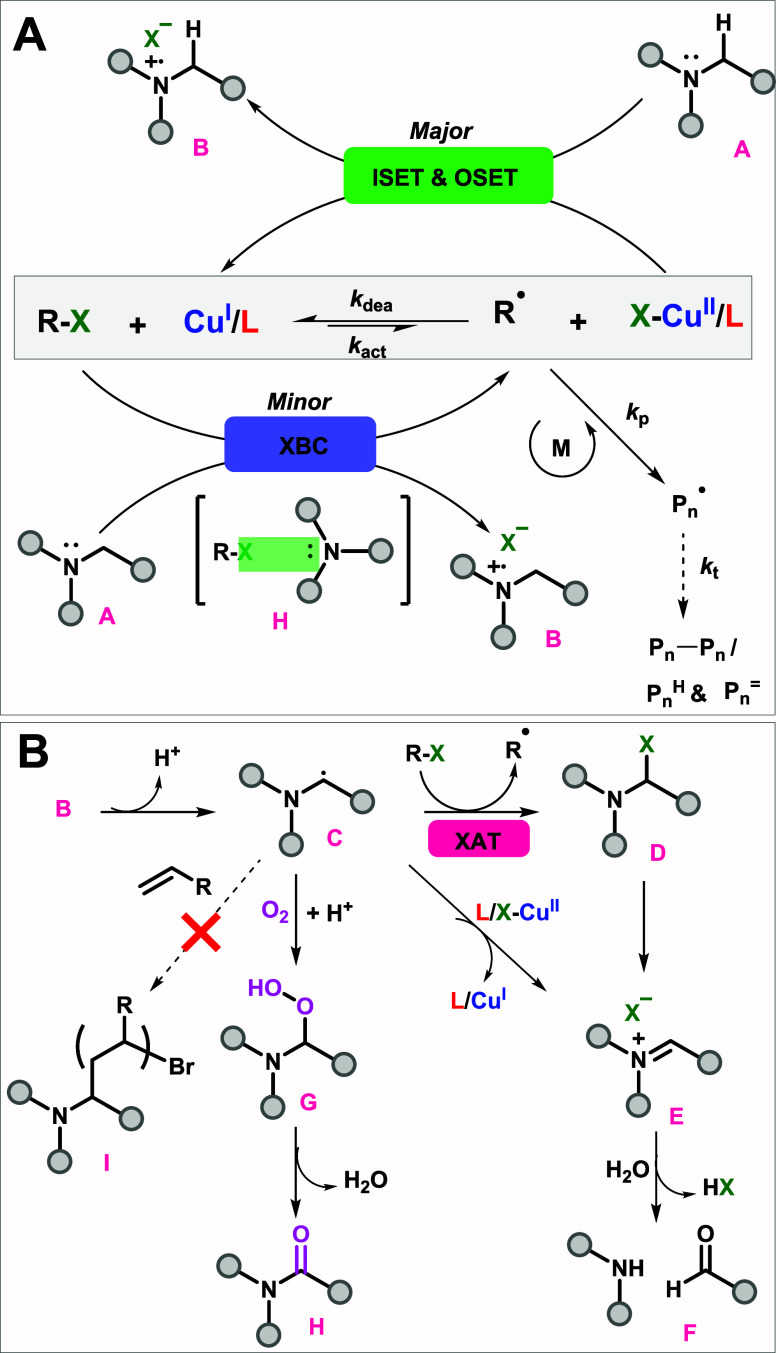
(A) General mechanism for ATRP in the presence of amines in the dark and (B) possible chemical events of oxidized amines in ATRP. ISET: inner sphere electron transfer. OSET: outer sphere electron transfer; XBC: halogen bonding complex; XAT: halogen atom transfer.

Additional control experiments were performed to identify other possible radical generation pathways. Without EBiB in the reaction medium, no polymerization was observed even at 60 °C in the presence of copper complexes (Br–Cu^II^/TPMA)^+^ Br^–^ or (Cu^II^/TPMA)^2+-^(OTf)_2_ and amines (e.g., Me_6_TREN, TPMA, PMDETA, DABCO), which suggests that EBiB was the sole source of initiating radicals (Figure 3b). Thus, aminoalkyl radicals formed as intermediates in the reduction process of Br–Cu^II^/L did not initiate polymerization (cf. Figure 4*infra*). Finally, no polymerization was observed without excess amines (only 1 equiv of TPMA vs CuBr_2_) or without EBiB and (Br–Cu^II^/ TPMA)^+^ Br^–^ at 60 °C, excluding the possibility of productive ligand-to-metal charge transfer (LMCT) of TPMA ligand to Cu^II^ and dissociation of Cu^II^/L complexes at 60 °C. (Figure 3c).^67,100,101^

### Overall Mechanistic Features of ATRP in the Presence of Amines

The general mechanism for amine-ARGET ATRP, or amine-SARA ATRP at higher temperatures, and the fate of amines after electron transfer reactions are illustrated in Figure 4. Amines (A) can play multiple roles in ATRP by acting as ligands, electron donors for the Cu^II^/L complex (reduction), or directly activating R-X at higher temperatures.^101^ Depending on the structure of the amine, the electron transfer (ET) to Cu^II^ may occur through OSET and/or ISET mechanism. The activation of R-X via the formation of a halogen bonding complex (XBC), although not very significant, could also facilitate activator regeneration and, thus, radical generation via an alternative pathway (SA). In all cases, the main role of amine is as a reducing agent, and it is only significant when it is used as a ligand in excess to Cu^II^ species. The reduction pathways resemble ARGET, where activators are (re)generated by electron transfer from the reducing agent. This facilitates a regenerative ATRP mechanism at <200 ppm levels of Cu. The kinetics and control of the ATRP of MA were simulated by Predici,^102^ using CuBr_2_/Me_6_TREN as the initial Cu- species and Me_6_TREN as a reducing agent (See Predici section, Figure S17–S18). The very small fraction (<0.1%) of Cu^I^/L activator is maintained at the steady state, confirming slow reduction for driving polymerization to completion.

The first oxidation products of amines (A) are amine radical cations (B). Deprotonation at the α-position of the amine radical cations, by e.g. free amines or potentially even intramolecularly, could generate α-aminoalkyl radicals (C), which are strong alkyl halide activators (halogen atom transfer, XAT).^103,104^ The radical (C) can be deactivated to form halogenated species (D) which could irreversibly rearrange to form iminium ions (E) and cannot efficiently initiate polymerization (I). Radical C can also be directly oxidized to cation E by Cu^II^/L species.^57^ Thus, one amine could reduce two Cu(II) species and form an iminium cation. Iminium cations may hydrolyze and form aldehydes (F) in the presence of water. The aldehydes were detected by ^1^H NMR by their distinctive peaks at 9.6 ppm in some reactions performed with amines (Figure S16). The oxygen tolerance provided by amines could be explained by 1) the ability of amines to form amides/formamides (H) (Figure S12).^89−91^ 2) Continuous generation of Cu^I^/L activators, and its reaction with O_2_ to form ROS (i.e., H_2_O_2_), which are scavenged by DMSO and form dimethyl sulfone (DMSO_2_),^26,105^ 3) reaction of O_2_ with radicals generated from R-X via either Cu^I^ or SA by amines, since R-X initiators are known to promote oxygen depletion in ATRP.^81,87^

## Conclusions

Various amines were investigated as reducing agents and supplemental activators in ATRP in the dark, and their predominant role as reducing agents was determined by kinetic experiments. Most important conclusions are 1) When used in excess in the dark, without light irradiation, all tested primary, secondary, and tertiary amines may act as a reducing agents (RA) for Cu^II^/L complex except for arylamines and heterocyclic aromatic amines; 2) Most tertiary amines reduce Cu^II^/L complex via OSET mechanism in agreement with their redox potentials, except DBU and TMG which react much faster than predicted by Marcus theory, suggesting an ISET process; 3) Changing (Br–Cu^II^/TPMA)^+^ Br^–^ to (Cu^II^/TPMA)^2+-^(OTf)_2_, a complex with more positive redox potential, accelerates the reduction of Cu^II^ and enables ATRP with many inexpensive amines; 4) Amines and the related products act as robust oxygen scavengers for ATRP, affording polymerization without degassing in vials with large head space relative to reaction mixture volume; 5) The polymerizations performed with amines were controlled as evidenced by linear semilogarithmic kinetic plots, good agreement of *M*_n,theo_ and *M*_n,GPC_ and clear evolution of GPC traces with time. 6) Ligand-like amines can directly activate R-X, especially at higher temperatures, providing additional radical sources, as supplemental activators (SA); 7) Radical cations formed from amines during reduction did not initiate new chains, forming noninitiating iminium cations, protonated amines, and other species. In summary, the results from this study contribute to the fundamental understanding of the electron transfer processes in ATRP in the presence of amines and enable the development of new and more universal reducing agents for ATRP. We also envisage broad applications of ARGET ATRP with amines considering their significantly improved workflow, oxygen tolerance, and commercial availability.

## Supporting Information Available

Details of experimental procedures and additional experiments and polymerization results; materials, instrumentation, experimentation, kinetics of polymerization, polymerization results with different targets of molecular weights, UV analyses on the stability of CuBr_2_ complexes, studied amines and the corresponding polymerization results, cyclic voltammetry, GPC trace results of the amines, complexes and Predici simulation (PDF)
