# Supplementary material for: Unraveling the Roles of Amines in Atom Transfer Radical Polymerization in the Dark
Source: J Am Chem Soc. 2025 Apr 2;147(15):12562–73. doi: 10.1021/jacs.4c18496 (PMC12006995; doi:10.1021/jacs.4c18496)
Supplement: Supplementary file 1 — ja4c18496_si_001.pdf [file ja4c18496_si_001.pdf]

Supporting Information

Unraveling the Roles of Amines in Atom Transfer Radical Polymerization in the Dark

Arman Moini Jazani, [#] Gorkem Yilmaz, [#] Mitchell Baumer, Julian Sobieski, Stefan Bernhard, Krzysztof Matyjaszewski*

AUTHOR ADDRESS

Department of Chemistry, Carnegie Mellon University, 4400 Fifth Avenue, Pittsburgh, Pennsylvania 15213, United States

Table of contents

Experimental Part……………………………………………………………………………………………..1

Materials………………………………………………………………………………………………1

Instrumentation…………………………………………………………..……………………………1

Procedures for polymerizations…………………………………………………………………………...3

Electrochemical properties and cyclic voltagramms……………………..…………………..…………………...5

$CuBr_2$/L reduction measurement by UV-VIS…………………………………………………..……………..15

Ligand exchange measurement by UV-VIS………….....………………….....……………………………………..17

GPC traces for polymerization with $CuBr_2$………………………………………………………………..……….18

Elimination reaction of R-Br with amines……………………………………………..………………….....19

Results of polymerization for ligand with impurities……………………………….……………………...……21

GPC traces for polymerization with $Cu(OTf)_2$…………………………………………………………...……...23

$Cu(OTf)_2$/L reduction measurement by UV-VIS………………………………………….....……………..…...24

GPC traces for oxygen tolerant polymerization……………………………………………………………25

Supplemental activation of alkyl chlorides…………………………………………………………………..28

Predici simulation……………………..…………………………………………………………………..30

References……………………………………………………………………………………………33

Experimental Part

Materials

Methyl acrylate (MA, 99%), copper(II) bromide ($CuBr_2$, 98%), ethyl 2-bromoisobutyrate (EBiB, 97%), dicyclohexylamine (DCHA, ≥99.9%), (±)-*trans*-1,2-diaminocyclohexane (DACH, 99%), dihexylamine (DHA, 97%), 2,2‘-bipyridyne (BPY, 99%), N,N-dimethylaminoethanol (DMAE, 99.5%), 2-(dimethylamino)ethyl

methacrylate (DMAEMA, 98%), triethanolamine (TEOA, 98%), N,N,N′,N″,N″-pentamethyldiethylenetriamine (PMDETA, 99%), N,N,N′,N′-tetramethylethylenediamine (TMED, 99%), 1,1,3,3-tetramethylguanidine (TMG, 99%), 1,4-diazabicyclo[2.2.2]octane (DABCO, ≥99%), 1,4,8,11-tetraazacyclotetradecane (CYC, 98%), acetonitrile (MeCN, ≥99.9%) from Sigma-Aldrich; piperidine (PIP, 99%) from Alfa Aesar; N,N-dicyclohexylmethylamine (DCHMA, 97%), pyrrolidine (PYR, 99%), copper(II) trifluoromethanesulfonate (triflate) ($Cu(OTf)_2$, 98%), 1,8-diazabicyclo[5.4.0]undec-7-ene (DBU, 98%) from Acros; tris(2-aminoethyl)amine (TREN, 97%) from Thermo Fisher Scientific; N,N,N′,N′-tetramethyl-p-phenylenediamine (TPMPD, >98%), phenothiazine (PTH, ≥98%) from Fluka; 4-methoxy-N,N-dimethylaniline (MDMA), tris(2-pyridylmethyl) amine (TPMA, 99%) and tris[2-(dimethylamino)ethyl]amine (Me_6TREN, 99%) from Ambeed; 1,3,5-trimethyl-1,3,5-triazinane (TMT, >98%), 1,1,4,7,10,10-hexamethyltriethylenetetramine (HMTETA, >98%), N-phenylglycine (PG, >97%), dimethyl sulfoxide (DMSO, >99%) from Tokyo Chemical Industry used as received.

MA was purified by passing through basic alumina. TPMA was recrystallized from hexane to remove all yellow impurities, yielding white crystals.[1]

Instrumentation

The apparent molecular weights ($M_{n,GPC}$) and dispersity ($Đ$) were measured relative to poly(methyl methacrylate) (PMMA) standards by gel permeation chromatography (GPC) conducted with a Waters 515 pump and Waters 2414 differential refractometer using PSS columns (SDV 10^5, 10^3, 500 Å) with THF as eluent at 35 °C and at a flow rate of 1 mL.min^{-1}. ^{1}H NMR spectra were collected using a Bruker Advance 500 MHz NMR spectrometer with d_6-DMSO as a solvent at room temperature.

UV-Vis-NIR spectra were recorded using an Agilent 8453 spectrophotometer.

Electrochemistry: Cyclic voltammetry was performed using a CH-Instrument Electrochemical Analyzer 600C potentiostat with a three-electrode system consisting of a silver wire pseudo-reference electrode, a platinum coil counter electrode, and a glassy carbon working electrode. Experiments were performed at 1 mM analyte concentration with 0.1 M tetra-*n*-butylammonium hexafluorophosphate as the supporting electrolyte in acetonitrile (MeCN) after purging the solution with argon until no traces of oxygen signals were visible in the CV. Voltammograms were collected using a scan rate of 0.1 V/s, , 0.5 V/s, and 1 V/s, and redox potentials were referenced to a ferrocene internal standard (E^0 (Fc^+/Fc) = 0.40 V vs saturated calomel electrode (SCE) in MeCN or a para-benzoquinone internal standard (E^0 (PBQ/PBQ^-) = -0.49 V vs SCE) in MeCN. PBQ was used as the internal standard when the analyte disrupted the ferrocene redox event.

Processing the voltammograms was done in two different ways, depending on whether the redox event was reversible or not. For reversible events, the peak positions corresponding to the oxidation and the reduction were averaged to find the half-wave potential of the event. This was then adjusted using a subsequent spectrum where ferrocene or p-benzoquinone was used as a reference standard by finding the difference between the

measured and the expected half-wave potential values of ferrocene, the reference compound (with respect to SCE) and applying the difference to the original voltammogram to reference it to SCE.

For irreversible events, processing began by fitting a polynomial equation to the region of the spectrum containing oxidation or reduction. For consistency with the literature, the oxidation peak position was found and reported. In addition, the inflection point of the modeled equation was determined using the x-intercept of the second derivative of the generated polynomial function. A tangent line to the model equation at the inflection point was also generated, by finding the slope of the modeled function at the inflection point and generating a linear function with the corresponding slope and the inflection coordinates as a point on the line. A section of the spectrum before the onset of oxidation was selected to model the baseline as a linear function, where the baseline and the aforementioned tangent line intersect were selected as the "onset point". These spectra were adjusted using ferrocene or p-benzoquinone as a reference standard, and the final values have been adjusted vs. SCE. The oxidation potentials of the amines to the amine radical cations are reported as reduction potential.

General procedure for polymerization with $(Br\text{-}Cu^{II}/TPMA)^+\ Br^-$ or $(Cu^{II}/TPMA)^{2+}\ {}^-(OTf)_2$ complex and amines

For a typical ATRP with amines, EBiB (56 mg, 0.29 mmol), MA (2.5 g, 29.0 mmol), $CuBr_2$ (1.3 mg, 5.8 µmol) or $Cu(OTf)_2$ (2.1 mg, 5.8 µmol), TPMA (1.7 mg, 5.8 µmol) were dissolved in DMSO in a volumetric flask (5 mL) and filled up with DMSO. The mixture was vortexed for 2 minutes. After transferring the solution (4 mL) to a glass vial (20 mL), amine (20 equiv of amines functional group in the structure to CuX_2/TPMA) was added to the polymerization mixture by a micropipette from a stock solution. The ratio of amines to $CuBr_2$ was adjusted to the functional groups of amines in the structure. For example, for ATRP with TREN which has 4 primary amines in the structure, only 5 equiv of TREN was used. The final concentrations were MA (5807 mM), EBiB (58.0 mM), $CuBr_2$ (1.16 mM) or $Cu(OTf)_2$ (1.16 mM), TPMA (1.16 mM) and DMSO (50% v/v).

The vial was sealed with a septum and deoxygenated by purging with N_2 gas for 10 mins. The polymerization reaction was left in a dark place for different times without stirring. The polymerization was stopped by opening the septum and transferring the polymerization solution to DMSO-d_6 and THF for ^{1}H-NMR and GPC characterization.

General procedure for oxygen-tolerant polymerization with $(Br\text{-}Cu^{II}/TPMA)^+\ Br^-$ or $(Cu^{II}/TPMA)^{2+}\ {}^-(OTf)_2$ complex and amines

For a typical ATRP with amines, EBiB (56 mg, 0.29 mmol), MA (2.5 g, 29 mmol), $CuBr_2$ (1.3 mg, 5.8 µmol) or $Cu(OTf)_2$ (2.1 mg, 5.8 µmol), TPMA (1.7 mg, 5.8 µmol) were dissolved in DMSO in a volumetric flask (5 mL) and filled up with DMSO. The mixture was vortexed for 2 minutes to mix all polymerization components. After transferring the solution (3 mL) to a glass vial (4 mL), amine (20 equiv of amines functional group in the structure to CuX_2/TPMA) was added to the polymerization mixture by a micropipette from a stock solution. The vial was capped and was left in a dark place for different times without stirring. The final concentrations were

MA (5807 mM), EBiB (58.0 mM), $CuBr_2$ (1.16 mM) or $Cu(OTf)_2$ (1.16 mM), TPMA (1.16 mM) and DMSO (50 % *v/v*).

The polymerization was stopped by transferring the polymerization solution to DMSO-d_6 and THF for ^{1}H-NMR and GPC characterization.

Procedure for polymerization with $(Br\text{-}Cu^{II}/Me_6TREN)^+Br^-$ complex and Me_6TREN

For a typical ATRP with amines, EBiB (56 mg, 0.29 mmol), MA (2.5 g, 29 mmol), $CuBr_2$ (1.3 mg, 5.8 µmol), Me_6TREN (1.3 mg, 5.8 µmol) were dissolved in DMSO in a volumetric flask (5 mL) and filled up with DMSO. The mixture was vortexed for 2 minutes. After transferring the solution (4 mL) to a glass vial (20 mL), additional Me_6TREN (5.4 mg, 23 µmol) was added to the polymerization mixture by a micropipette from a stock solution. The final concentrations were MA (5807 mM), EBiB (58.07 mM), $CuBr_2$ (1.16 mM), Me_6TREN (6.96 mM), and DMSO (50% *v/v*).

The vial was sealed with a septum and deoxygenated by purging with N_2 gas for 10 mins. The polymerization reaction was left in a dark place for different time without stirring. The polymerization was stopped by opening the septum and transferring the polymerization solution to DMSO-d_6 and THF for ^{1}H-NMR and GPC characterization.

Procedure for polymerization chain extension

Chain extension was performed by using the precursor PMA-Br (M_n = 9,250 g/mol, $Đ$ = 1.08), prepared by ATRP with $(Br\text{-}Cu^{II}/Me_6TREN)^+Br^-$ complex and Me_6TREN, as the macroinitiator. The conditions were adjusted as follows:

[PMA-Br]/[MA]/[$CuBr_2$]/[Me_6TREN]:1/200/0.1/0.5, [MA] = 5807 mM in DMSO. After the chemicals were taken in a Schlenk flask under N_2, the reaction vessel was kept in the dark for 4 h. Then, the polymerization mixture was precipitated in a methanol/water mixture twice to purify the chain-extended PMA (M_n = 12,700 g/mol, $Đ$ = 1.07) and analyzed by GPC.

Table S1. Redox potential (*peak*) of amines at different scan rates and kinetics and thermodynamic values of their OSET electron transfer with (Br-Cu^{II}/TPMA)$^+$ Br$^-$.[a]

Entry	Amine	$E_{red,amine}$ [a] (V vs SCE) (1.0 V/s)	$E_{red,amine}$ [a] (V vs SCE) (0.5 V/s)	$E_{red,amine}$ [a] (V vs SCE) (0.1 V/s)	ΔG (kJ·mol^{-1})	K_{eq}	$k_{red,theo}$ (mol·L^{-1}s^{-1})
1	BPY	2.38	2.22	2.38	252.9	4.5×10^{-45}	4.5×10^{-35}
2	DACH	2.04	2.00	1.91	220.2	2.5×10^{-39}	2.5×10^{-29}
3	TMG	1.32	1.30	1.28	150.7	3.8×10^{-27}	3.8×10^{-17}
4	PIP	1.27	1.23	1.20	145.9	2.7×10^{-26}	2.7×10^{-16}
5	DHA	1.14	1.14	1.15	133.3	4.2×10^{-24}	4.2×10^{-14}
6	DMAEMA	1.14	1.11	1.07	133.3	4.2×10^{-24}	4.2×10^{-14}
7	TMT	1.13	1.10	1.08	132.4	6.2×10^{-24}	6.2×10^{-14}
8	DBU	1.11	1.12	1.13	130.4	1.4×10^{-23}	1.4×10^{-13}
9	TPMA	1.06	1.05	1.03	125.6	9.5×10^{-23}	8.9×10^{-13}
10	PYR	1.00	0.99	0.96	119.8	9.8×10^{-22}	9.8×10^{-12}
11	PG	0.97	0.95	0.92	116.9	3.2×10^{-21}	3.2×10^{-11}
12	TMED	0.96	0.96	0.93	115.9	4.7×10^{-21}	4.7×10^{-11}
13	TEOA	0.91	0.91	0.87	111.1	3.3×10^{-20}	3.3×10^{-10}
14	DMAE	0.89	0.87	0.84	109.2	7.1×10^{-20}	7.1×10^{-10}
15	TEA	0.80	0.80	0.76	100.5	2.4×10^{-18}	2.4×10^{-8}
16	PTH[c]	0.71	0.70	0.68	-	-	-
17	DABCO[c]	0.68	0.67	0.67	-	-	-
18	HMTETA	0.68	0.67	0.65	88.9	2.5×10^{-16}	2.5×10^{-6}
19	PMDETA	0.66	0.66	0.63	87.0	5.5×10^{-16}	5.5×10^{-6}
20	DCHMA	0.64	0.63	0.60	85.1	1.2×10^{-15}	1.2×10^{-5}
21	MDMA	0.63	0.62	0.63	-	-	-
22	Me_6TREN[b]	0.62	0.62	0.60	91.7	8.6×10^{-17}	8.6×10^{-7}
23	TMPD[c]	0.22	0.21	0.22	-	-	-
24	TREN[d]	-	-	-	-	-	-
25	CYC[d]	-	-	-	-	-	-

[a] The reduction peak values calculated with respect to the SCE in MeCN, [b] Calculated based on $E_{1/2}$(Br-Cu^{II}/Me_6TREN)$^+$ $^-$Br = - 0.33 V. [c] Showed reversible process so only the half-wave potential of the reversible peak was reported. [d] Insoluble in MeCN.

Table S2. Redox potential *(inflection point)* of amines at different scan rates and kinetics and thermodynamic values of their OSET electron transfer with $(Br\text{-}Cu^{II}/TPMA)^{+}$ Br^{-}.[a]

Entry	Amine	$E_{red,amine}$ [a] (V vs SCE) (1.0 V/s)	$E_{red,amine}$ [a] (V vs SCE) (0.5 V/s)	$E_{red,amine}$ [a] (V vs SCE) (0.1 V/s)	ΔG (kJ·mol^{-1})	K_{eq}	$k_{red,theo}$ (mol·L^{-1}s^{-1})
1	BPY	2.04	2.13	2.02	220.2	2.5×10^{-39}	2.5×10^{-29}
2	DACH	1.27	1.30	1.29	145.9	2.7×10^{-26}	2.5×10^{-16}
3	TMG	1.25	1.23	1.21	143.9	5.8×10^{-26}	5.8×10^{-16}
4	PIP	1.16	1.13	1.11	135.3	1.9×10^{-34}	1.9×10^{-24}
5	TMT	1.05	1.02	1.01	124.6	1.4×10^{-22}	1.4×10^{-12}
6	DBU	1.04	1.04	1.06	123.7	2.1×10^{-22}	2.1×10^{-12}
7	DMAEMA	1.00	0.99	0.96	119.8	9.8×10^{-22}	9.8×10^{-12}
8	DHA	0.98	1.01	1.01	117.9	2.1×10^{-21}	2.1×10^{-11}
9	TPMA	0.98	0.97	0.96	117.9	2.1×10^{-21}	2.1×10^{-11}
10	PG	0.92	0.90	0.87	112.1	2.2×10^{-20}	2.2×10^{-10}
11	TMED	0.83	0.83	0.82	103.4	7.4×10^{-19}	7.4×10^{-9}
12	TEOA	0.81	0.80	0.78	101.5	1.6×10^{-18}	1.6×10^{-8}
13	PYR	0.77	0.78	0.80	97.6	7.6×10^{-18}	7.6×10^{-8}
14	DMAE	0.75	0.74	0.73	95.7	1.7×10^{-17}	1.7×10^{-7}
15	TEA	0.69	0.70	0.66	89.9	1.7×10^{-16}	1.7×10^{-6}
16	PTH	0.64	0.63	0.61	85.1	1.2×10^{-15}	1.2×10^{-5}
17	DABCO	0.62	0.61	0.61	83.2	2.6×10^{-15}	2.6×10^{-5}
18	HMTETA	0.58	0.57	0.56	79.3	1.2×10^{-14}	1.2×10^{-4}
19	DCHMA	0.56	0.55	0.53	117.9	2.1×10^{-21}	2.1×10^{-11}
20	PMDETA	0.56	0.56	0.54	77.4	2.7×10^{-14}	2.7×10^{-4}
21	MDMA	0.56	0.56	0.54	77.4	2.7×10^{-14}	2.7×10^{-4}
22	Me_6TREN[b]	0.54	0.54	0.53	83.9	1.9×10^{-15}	1.9×10^{-5}
23	TMPD	0.15	0.14	0.13	37.8	2.3×10^{-7}	2,345
24	TREN[c]	-	-	-	-	-	-
25	CYC[c]	-	-	-	-	-	-

[a] The reduction values calculated with respect to the SCE in MeCN, [b] Calculated based on $E_{1/2}(Br\text{-}Cu^{II}/Me_6TREN)^{+}$ ^{-}Br = - 0.33 V. [c] Insoluble in MeCN.

Table S3. Redox potential *(onset)* of amines at different scan rates and kinetics and thermodynamic values of their OSET electron transfer with $(Br\text{-}Cu^{II}/TPMA)^+$ Br^-. [a]

Entry	Amine	$E_{red,amine}$ [a] (V vs SCE) (1.0 V/s)	$E_{red,amine}$ [a] (V vs SCE) (0.5 V/s)	$E_{red,amine}$ [a] (V vs SCE) (0.1 V/s)	ΔG ($kJ\cdot mol^{-1}$)	K_{eq}	$k_{red,theo}$ ($mol\cdot L^{-1}s^{-1}$)
1	BPY	1.64	1.91	1.69	181.6	1.5×10^{-32}	1.5×10^{-22}
2	TMG	1.15	1.15	1.15	134.3	2.9×10^{-24}	2.9×10^{-14}
3	DACH	1.02	1.03	0.97	121.8	4.5×10^{-22}	4.5×10^{-22}
4	TMT	0.97	0.95	0.95	117.0	3.2×10^{-21}	3.2×10^{-11}
5	DBU	0.92	0.94	0.95	112.1	2.2×10^{-20}	2.8×10^{-10}
6	DMAEMA	0.91	0.90	0.88	111.1	3.3×10^{-20}	3.3×10^{-10}
7	TPMA	0.91	0.91	0.90	111.1	3.3×10^{-20}	3.3×10^{-10}
8	PG	0.86	0.85	0.83	106.3	2.3×10^{-19}	2.3×10^{-9}
9	DHA	0.84	0.86	0.86	104.4	5.0×10^{-19}	5.0×10^{-9}
10	PIP	0.82	0.94	0.99	102.5	1.1×10^{-18}	1.1×10^{-8}
11	TMED	0.73	0.74	0.73	93.8	3.6×10^{-17}	3.6×10^{-7}
12	TEOA	0.72	0.72	0.68	92.8	5.4×10^{-17}	5.4×10^{-7}
13	DMAE	0.66	0.64	0.64	87.0	5.5×10^{-16}	5.5×10^{-6}
14	PYR	0.62	0.64	0.64	83.2	2.6×10^{-15}	2.6×10^{-5}
15	TEA	0.59	0.61	0.59	80.3	8.5×10^{-15}	8.5×10^{-5}
16	PTH[c]	0.58	0.57	0.57	-	-	-
17	DABCO[c]	0.57	0.57	0.57	-	-	-
18	MDMA[c]	0.52	0.52	0.49	-	-	-
19	DCHMA	0.50	0.49	0.47	71.6	2.8×10^{-13}	2.8×10^{-3}
20	HMTETA	0.50	0.50	0.49	71.6	2.8×10^{-13}	2.8×10^{-3}
21	PMDETA	0.49	0.48	0.47	70.6	4.2×10^{-13}	4.2×10^{-3}
22	Me_6TREN[b]	0.45	0.46	0.45	75.3	6.4×10^{-14}	6.4×10^{-4}
23	TMPD[c]	0.10	0.09	0.07	-	-	-
24	TREN[d]	-	-	-	-	-	-
25	CYC[d]	-	-	-	-	-	-

[a] Values calculated with respect to the SCE in MeCN. [b] Calculated based on $E_{1/2}(Br\text{-}Cu^{II}/Me_6TREN)^{+}$ ^{-}Br = - 0.33 V. [c] Showed a reversible process, so only the half-wave potential of the reversible peak was reported. [d] Insoluble in MeCN.

Table S4. Redox potential *(half wave potential*) of reversible amines at different scan rates and kinetics and thermodynamic values of their OSET electron transfer with $(Br\text{-}Cu^{II}/TPMA)^{+}\ Br^{-}$.[a]

Entry	Amine	$E_{1/2}$ [a] (V vs SCE) (1.0 V/s)	$E_{1/2}$ [a] (V vs SCE) (0.5 V/s)	$E_{1/2}$ [a] (V vs SCE) (0.1 V/s)	ΔG (kJ·mol^{-1})	K_{eq}	$k_{red,theo}$ (mol·L^{-1}s^{-1})
1	PTH	0.66	0.65	0.65	87.0	5.5×10^{-16}	5.5×10^{-6}
2	DABCO	0.64	0.63	-	85.1	1.2×10^{-15}	1.2×10^{-5}
3	MDMA	0.60	0.61	0.62	81.2	5.7×10^{-15}	5.7×10^{-5}
4	TMPD	0.19	0.18	0.20	41.7	4.9×10^{-8}	493.9

[a] Values calculated with respect to the SCE in MeCN.

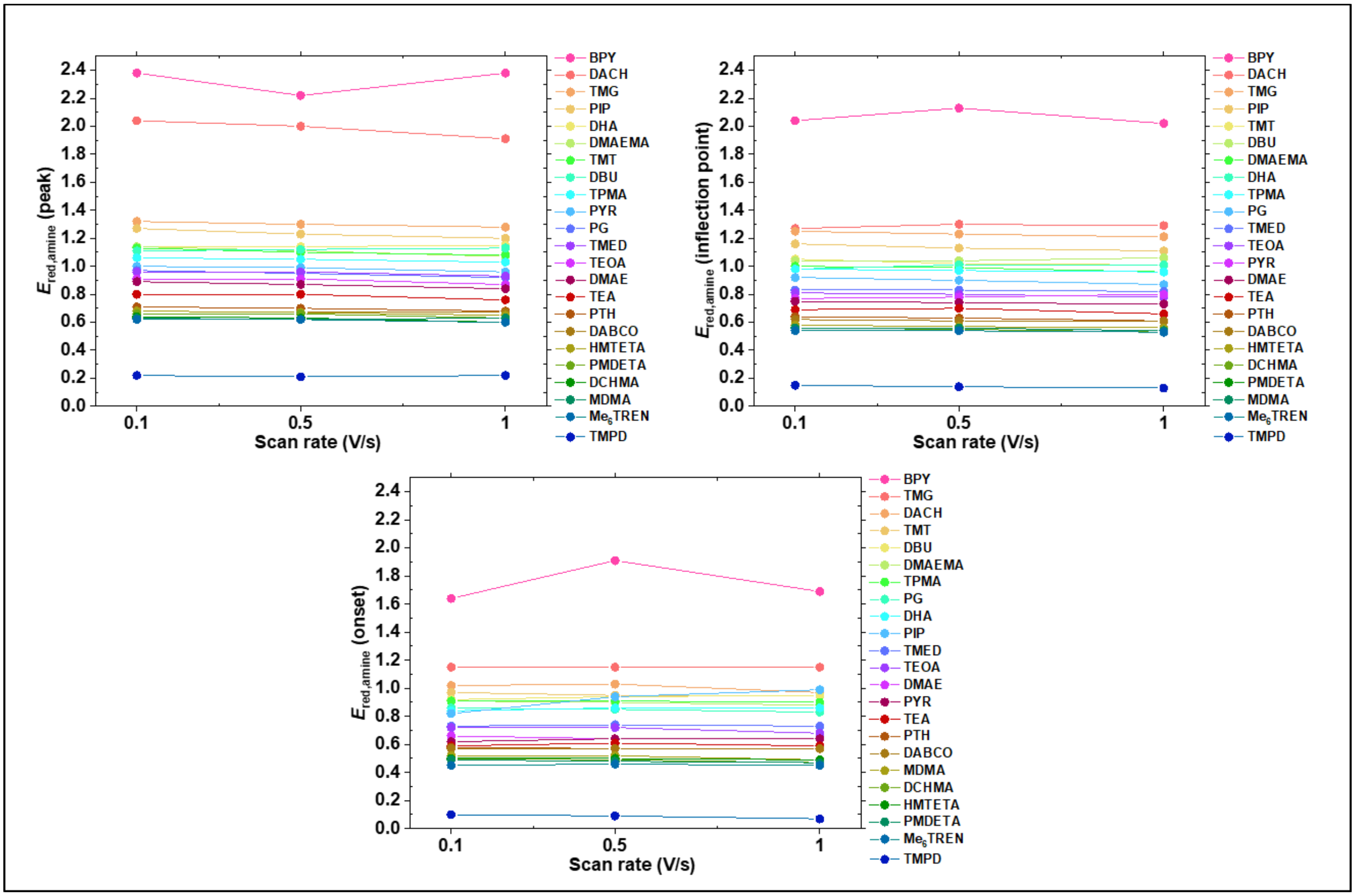

Figure S1. $E_{red,amine}$ at peak (top left), inflection point (top right) and onset (bottom) versus scan rates with respect to the SCE in MeCN.

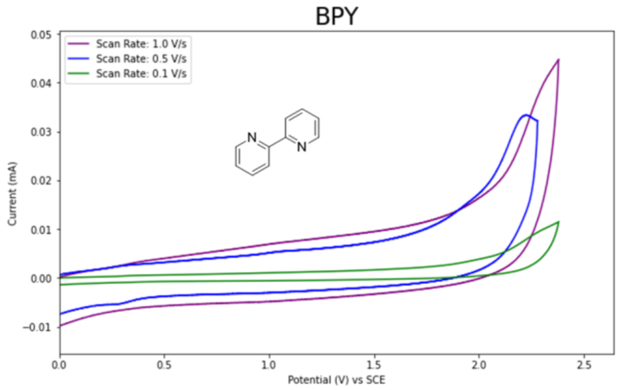
BPY
Scan Rate: 1.0 V/s
Scan Rate: 0.5 V/s
Scan Rate: 0.1 V/s
Current (mA)
Potential (V) vs SCE

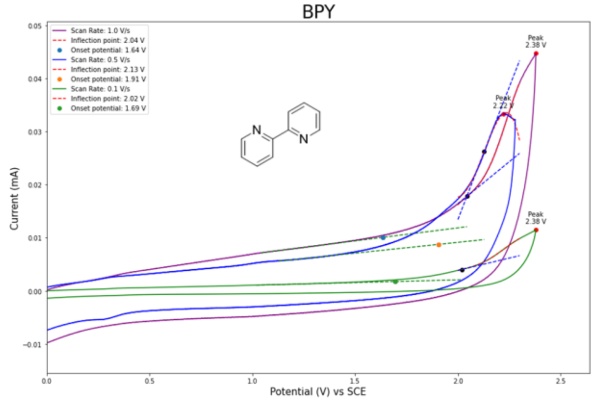
BPY
Scan Rate: 1.0 V/s
Inflection point: 2.04 V
Onset potential: 1.64 V
Scan Rate: 0.5 V/s
Inflection point: 2.13 V
Onset potential: 1.91 V
Scan Rate: 0.1 V/s
Inflection point: 2.02 V
Onset potential: 1.69 V
Peak 2.38 V
Peak 2.22 V
Peak 2.38 V
Current (mA)
Potential (V) vs SCE

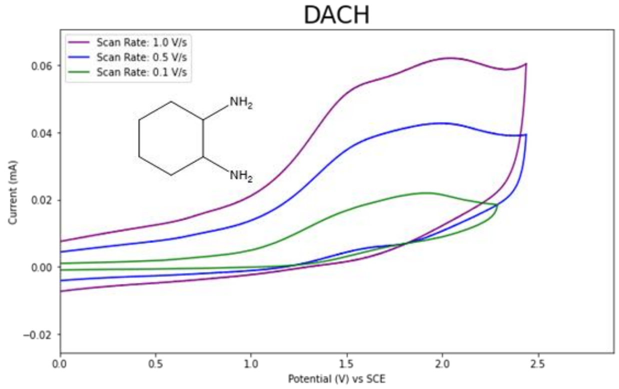
DACH
Scan Rate: 1.0 V/s
Scan Rate: 0.5 V/s
Scan Rate: 0.1 V/s
NH2
NH2
Current (mA)
Potential (V) vs SCE

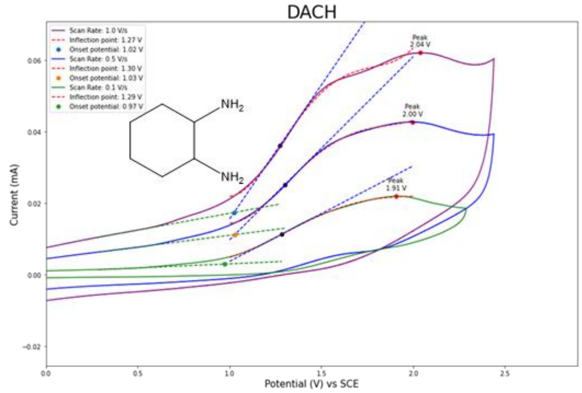
DACH
Scan Rate: 1.0 V/s
Inflection point: 1.27 V
Onset potential: 1.02 V
Scan Rate: 0.5 V/s
Inflection point: 1.30 V
Onset potential: 1.03 V
Scan Rate: 0.1 V/s
Inflection point: 1.29 V
Onset potential: 0.97 V
Peak 2.04 V
Peak 2.00 V
Peak 1.91 V
NH2
NH2
Current (mA)
Potential (V) vs SCE

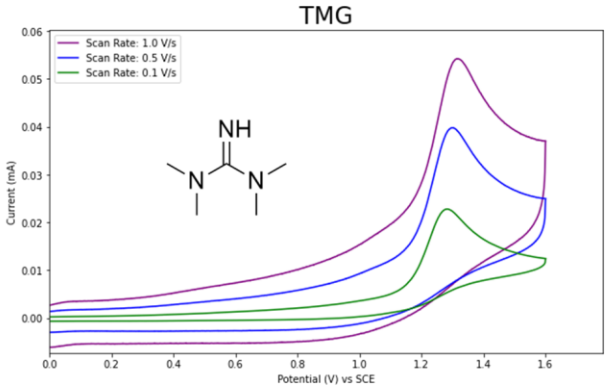
TMG
Scan Rate: 1.0 V/s
Scan Rate: 0.5 V/s
Scan Rate: 0.1 V/s
NH
N
N
Current (mA)
Potential (V) vs SCE

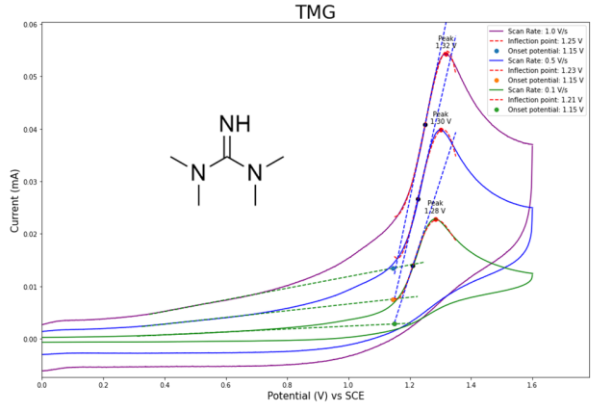
TMG
Scan Rate: 1.0 V/s
Inflection point: 1.25 V
Onset potential: 1.15 V
Scan Rate: 0.5 V/s
Inflection point: 1.23 V
Onset potential: 1.15 V
Scan Rate: 0.1 V/s
Inflection point: 1.21 V
Onset potential: 1.15 V
Peak 1.32 V
Peak 1.30 V
Peak 1.28 V
NH
N
N
Current (mA)
Potential (V) vs SCE

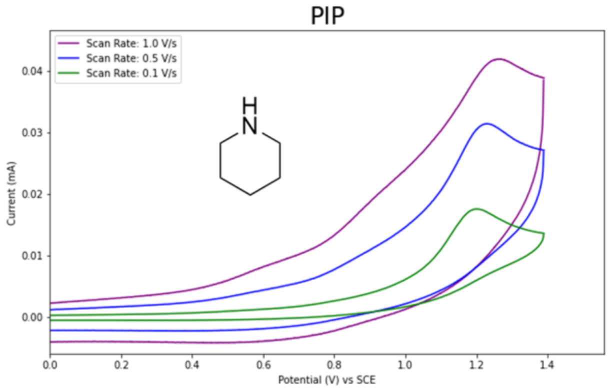
PIP
Scan Rate: 1.0 V/s
Scan Rate: 0.5 V/s
Scan Rate: 0.1 V/s
H
N
Current (mA)
Potential (V) vs SCE

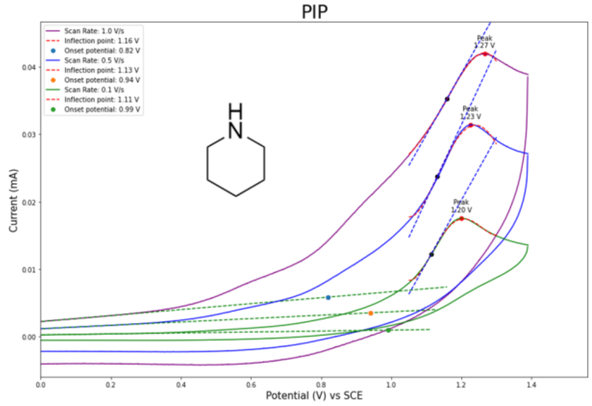
PIP
Scan Rate: 1.0 V/s
Inflection point: 1.16 V
Onset potential: 0.82 V
Scan Rate: 0.5 V/s
Inflection point: 1.13 V
Onset potential: 0.94 V
Scan Rate: 0.1 V/s
Inflection point: 1.11 V
Onset potential: 0.99 V
Peak 1.27 V
Peak 1.23 V
Peak 1.20 V
H
N
Current (mA)
Potential (V) vs SCE

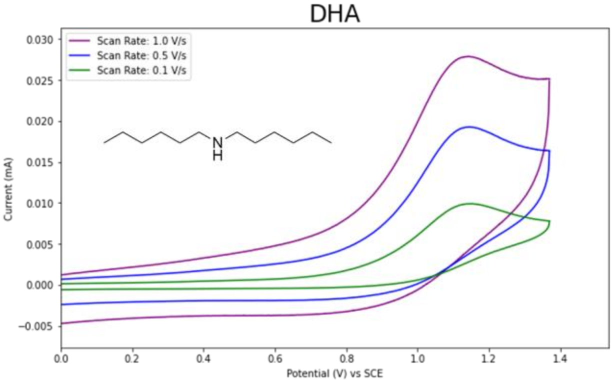
DHA
Scan Rate: 1.0 V/s
Scan Rate: 0.5 V/s
Scan Rate: 0.1 V/s
N
H
Current (mA)
Potential (V) vs SCE

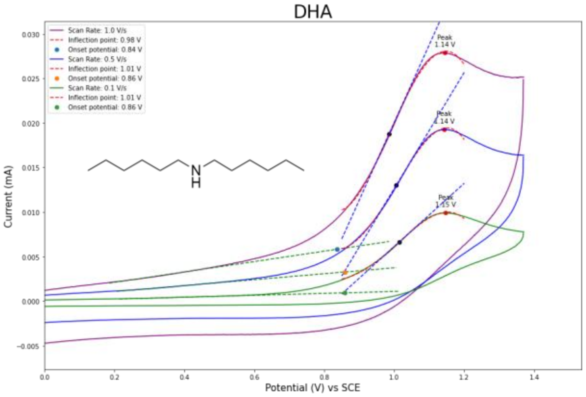
DHA
Scan Rate: 1.0 V/s
Inflection point: 0.98 V
Onset potential: 0.84 V
Scan Rate: 0.5 V/s
Inflection point: 1.01 V
Onset potential: 0.86 V
Scan Rate: 0.1 V/s
Inflection point: 1.01 V
Onset potential: 0.86 V
Peak
1.14 V
Peak
1.14 V
Peak
1.15 V
N
H
Current (mA)
Potential (V) vs SCE

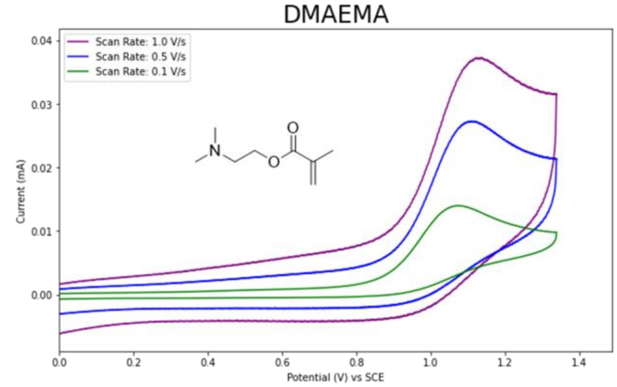
DMAEMA
Scan Rate: 1.0 V/s
Scan Rate: 0.5 V/s
Scan Rate: 0.1 V/s
N
O
O
Current (mA)
Potential (V) vs SCE

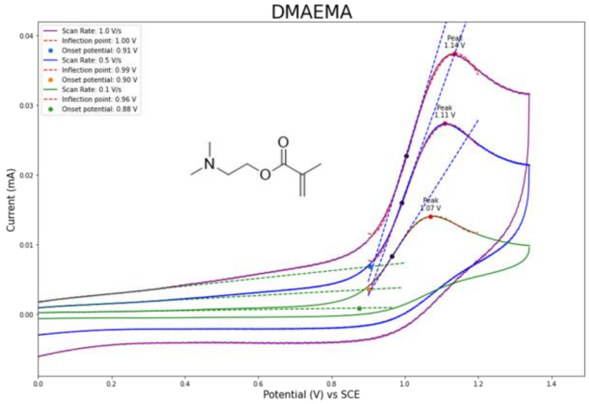
DMAEMA
Scan Rate: 1.0 V/s
Inflection point: 1.00 V
Onset potential: 0.91 V
Scan Rate: 0.5 V/s
Inflection point: 0.99 V
Onset potential: 0.90 V
Scan Rate: 0.1 V/s
Inflection point: 0.96 V
Onset potential: 0.88 V
Peak
1.14 V
Peak
1.11 V
Peak
1.07 V
N
O
O
Current (mA)
Potential (V) vs SCE

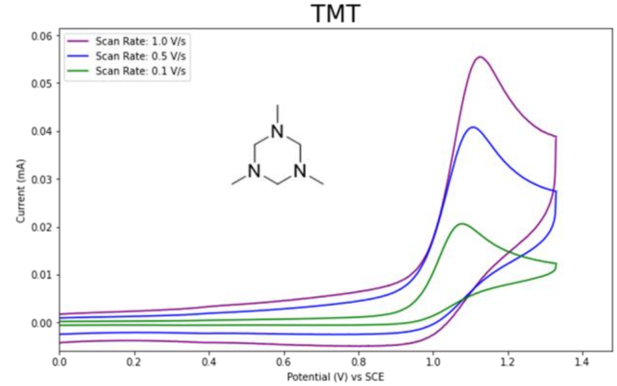
TMT
Scan Rate: 1.0 V/s
Scan Rate: 0.5 V/s
Scan Rate: 0.1 V/s
N
N
N
Current (mA)
Potential (V) vs SCE

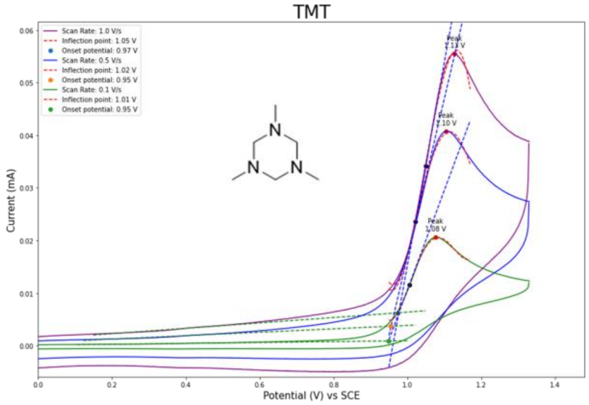
TMT
Scan Rate: 1.0 V/s
Inflection point: 1.05 V
Onset potential: 0.97 V
Scan Rate: 0.5 V/s
Inflection point: 1.02 V
Onset potential: 0.95 V
Scan Rate: 0.1 V/s
Inflection point: 1.01 V
Onset potential: 0.95 V
Peak
1.13 V
Peak
1.10 V
Peak
1.08 V
N
N
N
Current (mA)
Potential (V) vs SCE

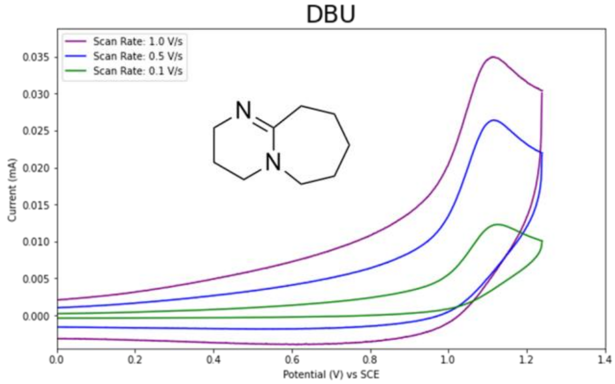
DBU
Scan Rate: 1.0 V/s
Scan Rate: 0.5 V/s
Scan Rate: 0.1 V/s
N
N
Current (mA)
Potential (V) vs SCE

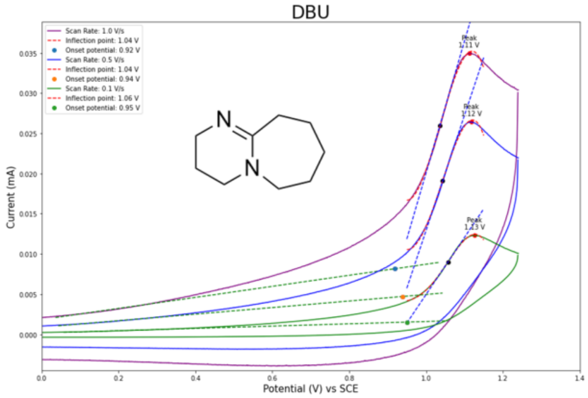
DBU
Scan Rate: 1.0 V/s
Inflection point: 1.04 V
Onset potential: 0.92 V
Scan Rate: 0.5 V/s
Inflection point: 1.04 V
Onset potential: 0.94 V
Scan Rate: 0.1 V/s
Inflection point: 1.06 V
Onset potential: 0.95 V
Peak
1.11 V
Peak
1.12 V
Peak
1.13 V
N
N
Current (mA)
Potential (V) vs SCE

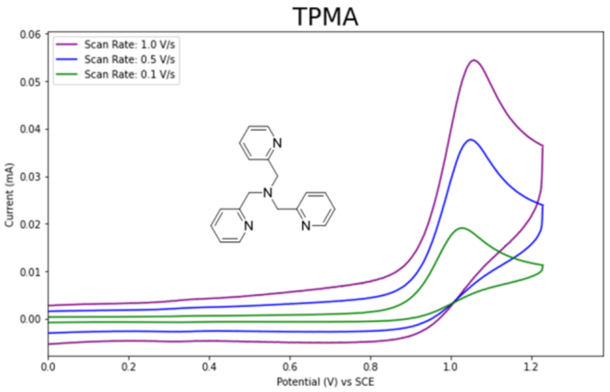
TPMA
Scan Rate: 1.0 V/s
Scan Rate: 0.5 V/s
Scan Rate: 0.1 V/s
Current (mA)
Potential (V) vs SCE

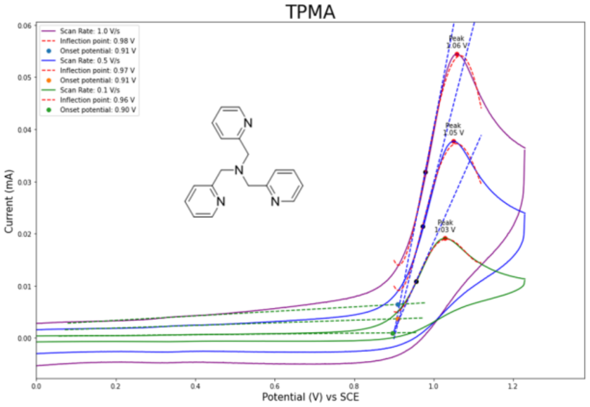
TPMA
Scan Rate: 1.0 V/s
Inflection point: 0.98 V
Onset potential: 0.91 V
Scan Rate: 0.5 V/s
Inflection point: 0.97 V
Onset potential: 0.91 V
Scan Rate: 0.1 V/s
Inflection point: 0.96 V
Onset potential: 0.90 V
Peak 1.06 V
Peak 1.05 V
Peak 1.03 V
Current (mA)
Potential (V) vs SCE

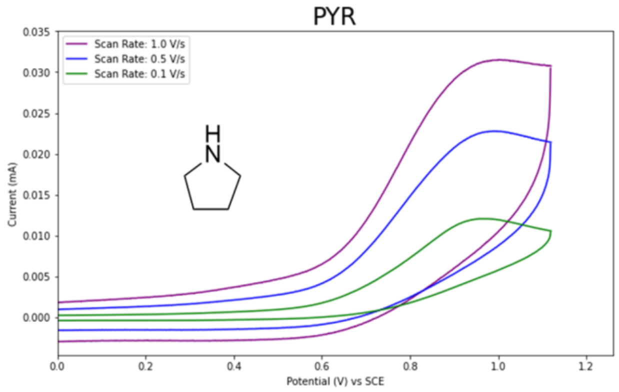
PYR
Scan Rate: 1.0 V/s
Scan Rate: 0.5 V/s
Scan Rate: 0.1 V/s
Current (mA)
Potential (V) vs SCE

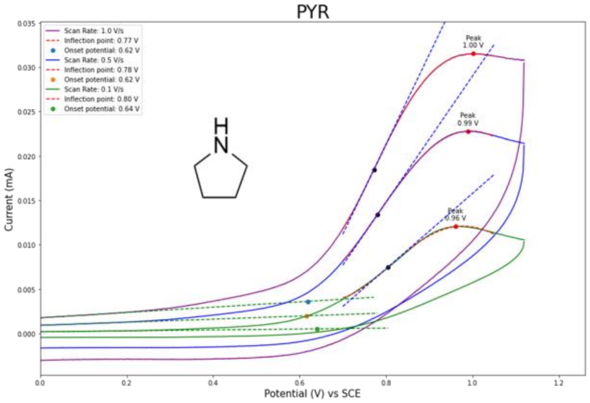
PYR
Scan Rate: 1.0 V/s
Inflection point: 0.77 V
Onset potential: 0.62 V
Scan Rate: 0.5 V/s
Inflection point: 0.78 V
Onset potential: 0.62 V
Scan Rate: 0.1 V/s
Inflection point: 0.80 V
Onset potential: 0.64 V
Peak 1.00 V
Peak 0.99 V
Peak 0.96 V
Current (mA)
Potential (V) vs SCE

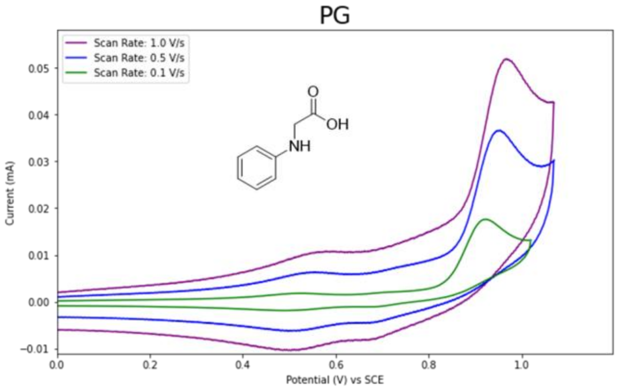
PG
Scan Rate: 1.0 V/s
Scan Rate: 0.5 V/s
Scan Rate: 0.1 V/s
Current (mA)
Potential (V) vs SCE

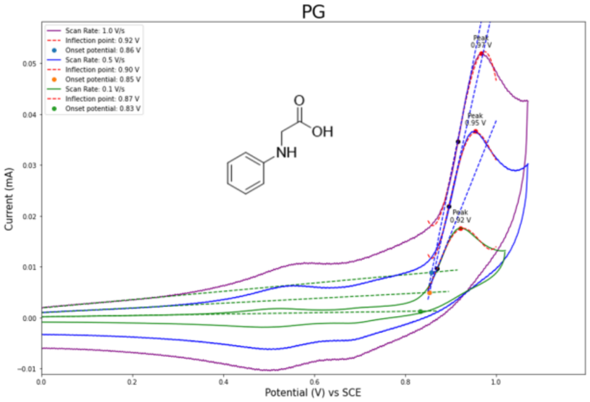
PG
Scan Rate: 1.0 V/s
Inflection point: 0.92 V
Onset potential: 0.86 V
Scan Rate: 0.5 V/s
Inflection point: 0.90 V
Onset potential: 0.85 V
Scan Rate: 0.1 V/s
Inflection point: 0.87 V
Onset potential: 0.83 V
Peak 0.95 V
Peak 0.92 V
Current (mA)
Potential (V) vs SCE

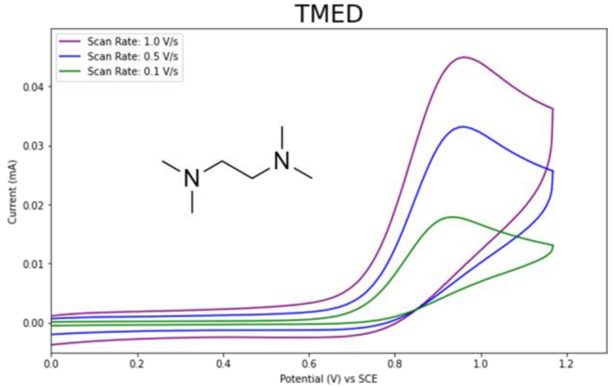
TMED
Scan Rate: 1.0 V/s
Scan Rate: 0.5 V/s
Scan Rate: 0.1 V/s
Current (mA)
Potential (V) vs SCE

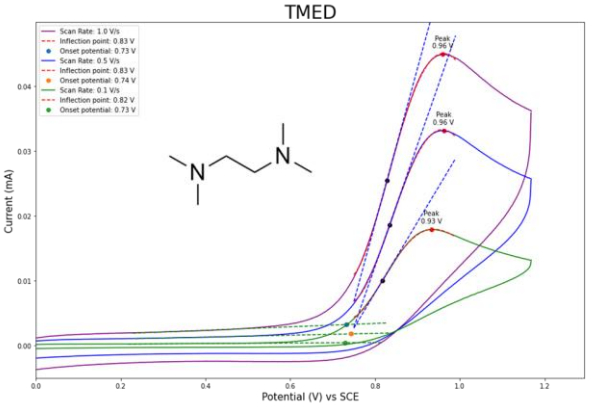
TMED
Scan Rate: 1.0 V/s
Inflection point: 0.83 V
Onset potential: 0.73 V
Scan Rate: 0.5 V/s
Inflection point: 0.83 V
Onset potential: 0.74 V
Scan Rate: 0.1 V/s
Inflection point: 0.82 V
Onset potential: 0.73 V
Peak 0.96 V
Peak 0.96 V
Peak 0.93 V
Current (mA)
Potential (V) vs SCE

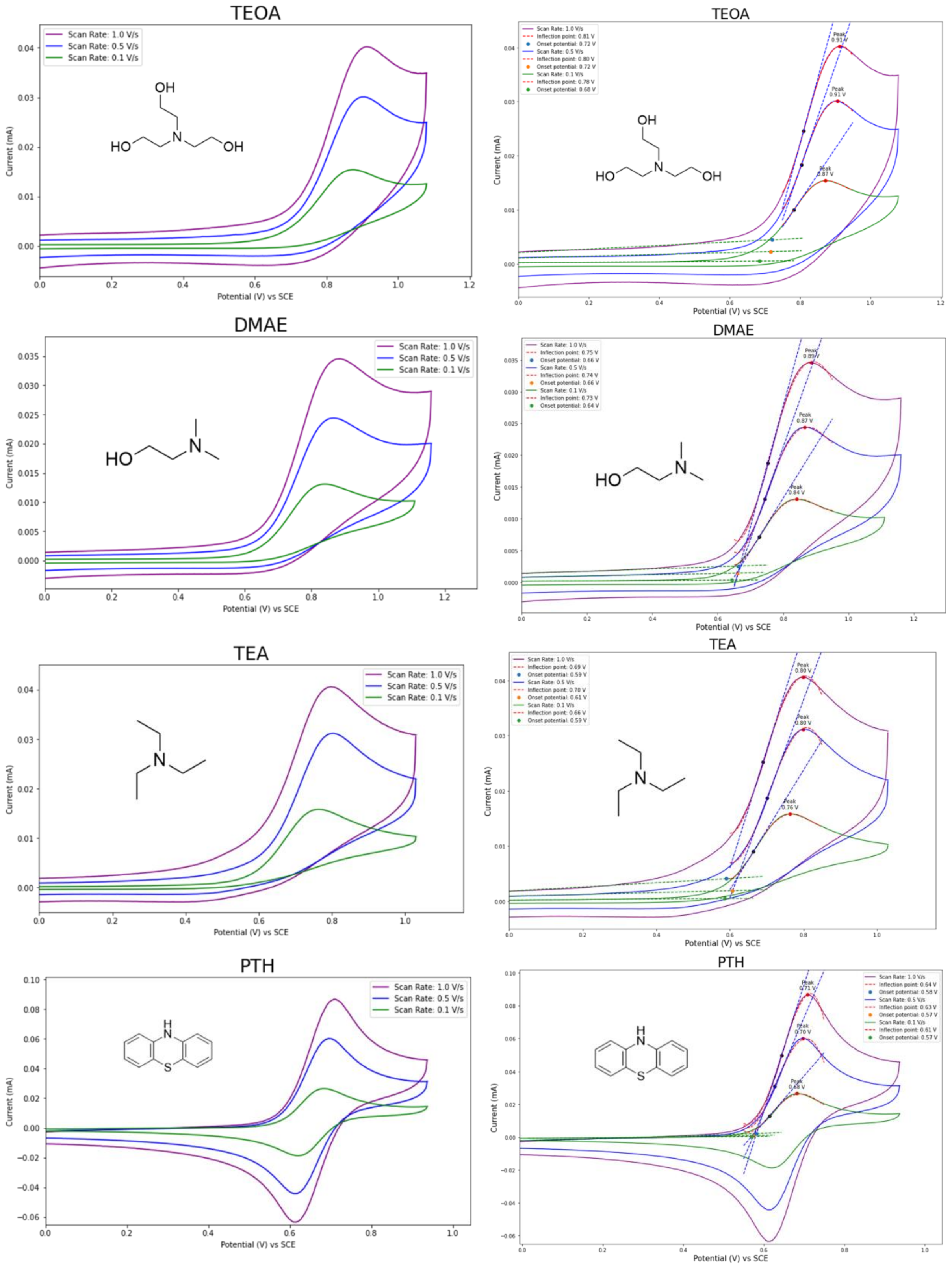
TEOA
Scan Rate: 1.0 V/s
Scan Rate: 0.5 V/s
Scan Rate: 0.1 V/s
Current (mA)
Potential (V) vs SCE
TEOA
Scan Rate: 1.0 V/s
Inflection point: 0.81 V
Onset potential: 0.72 V
Scan Rate: 0.5 V/s
Inflection point: 0.80 V
Onset potential: 0.72 V
Scan Rate: 0.1 V/s
Inflection point: 0.78 V
Onset potential: 0.68 V
Peak 0.91 V
Peak 0.91 V
Peak 0.87 V
DMAE
Scan Rate: 1.0 V/s
Scan Rate: 0.5 V/s
Scan Rate: 0.1 V/s
DMAE
Scan Rate: 1.0 V/s
Inflection point: 0.75 V
Onset potential: 0.66 V
Scan Rate: 0.5 V/s
Inflection point: 0.74 V
Onset potential: 0.66 V
Scan Rate: 0.1 V/s
Inflection point: 0.73 V
Onset potential: 0.64 V
Peak 0.89 V
Peak 0.87 V
Peak 0.84 V
TEA
Scan Rate: 1.0 V/s
Scan Rate: 0.5 V/s
Scan Rate: 0.1 V/s
TEA
Scan Rate: 1.0 V/s
Inflection point: 0.69 V
Onset potential: 0.59 V
Scan Rate: 0.5 V/s
Inflection point: 0.70 V
Onset potential: 0.61 V
Scan Rate: 0.1 V/s
Inflection point: 0.66 V
Onset potential: 0.59 V
Peak 0.80 V
Peak 0.80 V
Peak 0.76 V
PTH
Scan Rate: 1.0 V/s
Scan Rate: 0.5 V/s
Scan Rate: 0.1 V/s
PTH
Scan Rate: 1.0 V/s
Inflection point: 0.64 V
Onset potential: 0.58 V
Scan Rate: 0.5 V/s
Inflection point: 0.63 V
Onset potential: 0.57 V
Scan Rate: 0.1 V/s
Inflection point: 0.61 V
Onset potential: 0.57 V
Peak 0.71 V
Peak 0.70 V
Peak 0.68 V

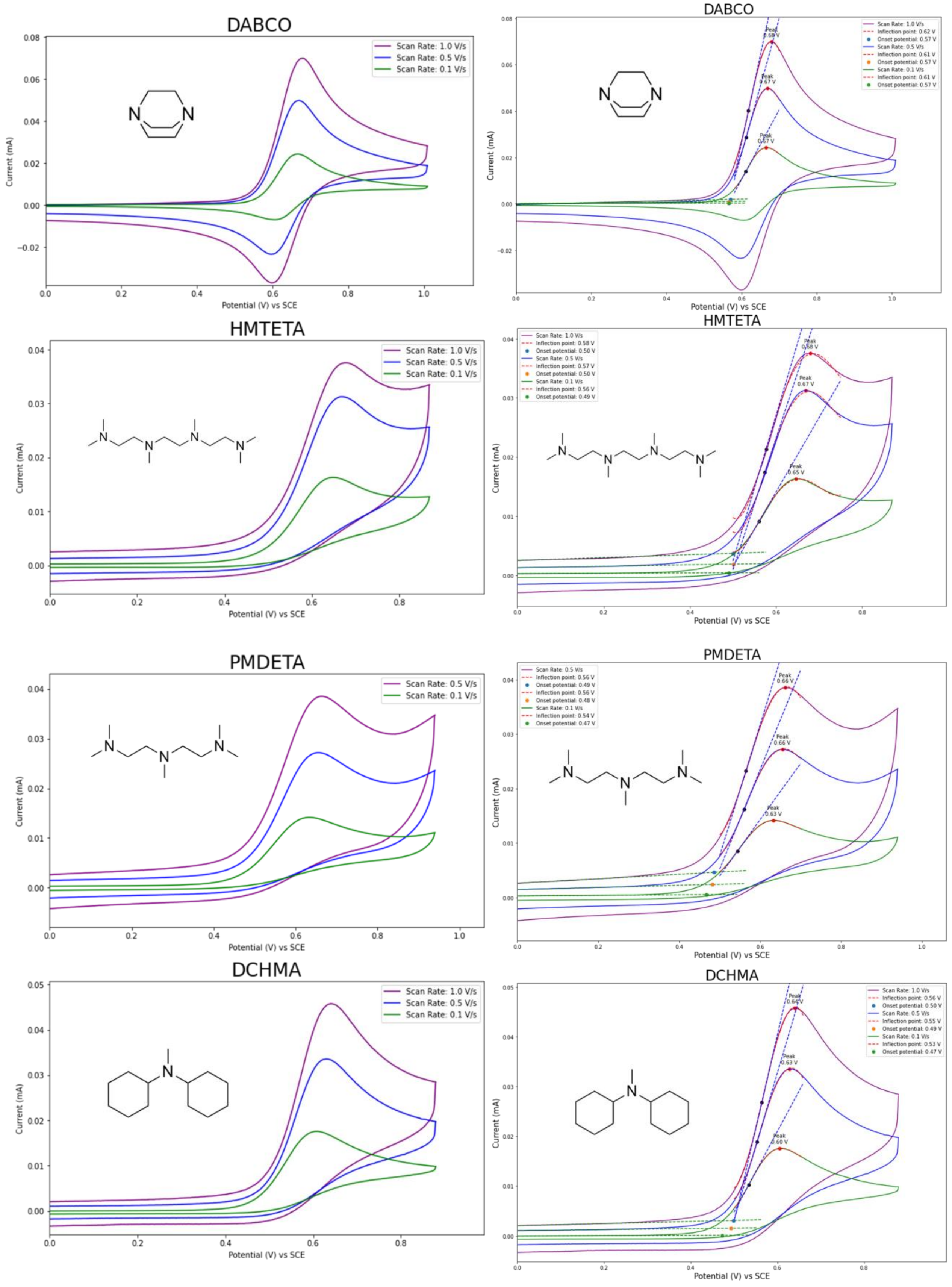
DABCO
Scan Rate: 1.0 V/s
Scan Rate: 0.5 V/s
Scan Rate: 0.1 V/s
Current (mA)
Potential (V) vs SCE
N
N
DABCO
Scan Rate: 1.0 V/s
Inflection point: 0.62 V
Onset potential: 0.57 V
Scan Rate: 0.5 V/s
Inflection point: 0.61 V
Onset potential: 0.57 V
Scan Rate: 0.1 V/s
Inflection point: 0.61 V
Onset potential: 0.57 V
Peak 0.67 V
HMTETA
Scan Rate: 1.0 V/s
Scan Rate: 0.5 V/s
Scan Rate: 0.1 V/s
HMTETA
Scan Rate: 1.0 V/s
Inflection point: 0.58 V
Onset potential: 0.50 V
Scan Rate: 0.5 V/s
Inflection point: 0.57 V
Onset potential: 0.50 V
Scan Rate: 0.1 V/s
Inflection point: 0.56 V
Onset potential: 0.49 V
Peak 0.68 V
Peak 0.67 V
Peak 0.65 V
PMDETA
Scan Rate: 0.5 V/s
Scan Rate: 0.1 V/s
PMDETA
Scan Rate: 0.5 V/s
Inflection point: 0.56 V
Onset potential: 0.49 V
Inflection point: 0.56 V
Onset potential: 0.48 V
Scan Rate: 0.1 V/s
Inflection point: 0.54 V
Onset potential: 0.47 V
Peak 0.66 V
Peak 0.66 V
Peak 0.63 V
DCHMA
Scan Rate: 1.0 V/s
Scan Rate: 0.5 V/s
Scan Rate: 0.1 V/s
DCHMA
Scan Rate: 1.0 V/s
Inflection point: 0.56 V
Onset potential: 0.50 V
Scan Rate: 0.5 V/s
Inflection point: 0.55 V
Onset potential: 0.49 V
Scan Rate: 0.1 V/s
Inflection point: 0.53 V
Onset potential: 0.47 V
Peak 0.64 V
Peak 0.63 V
Peak 0.60 V

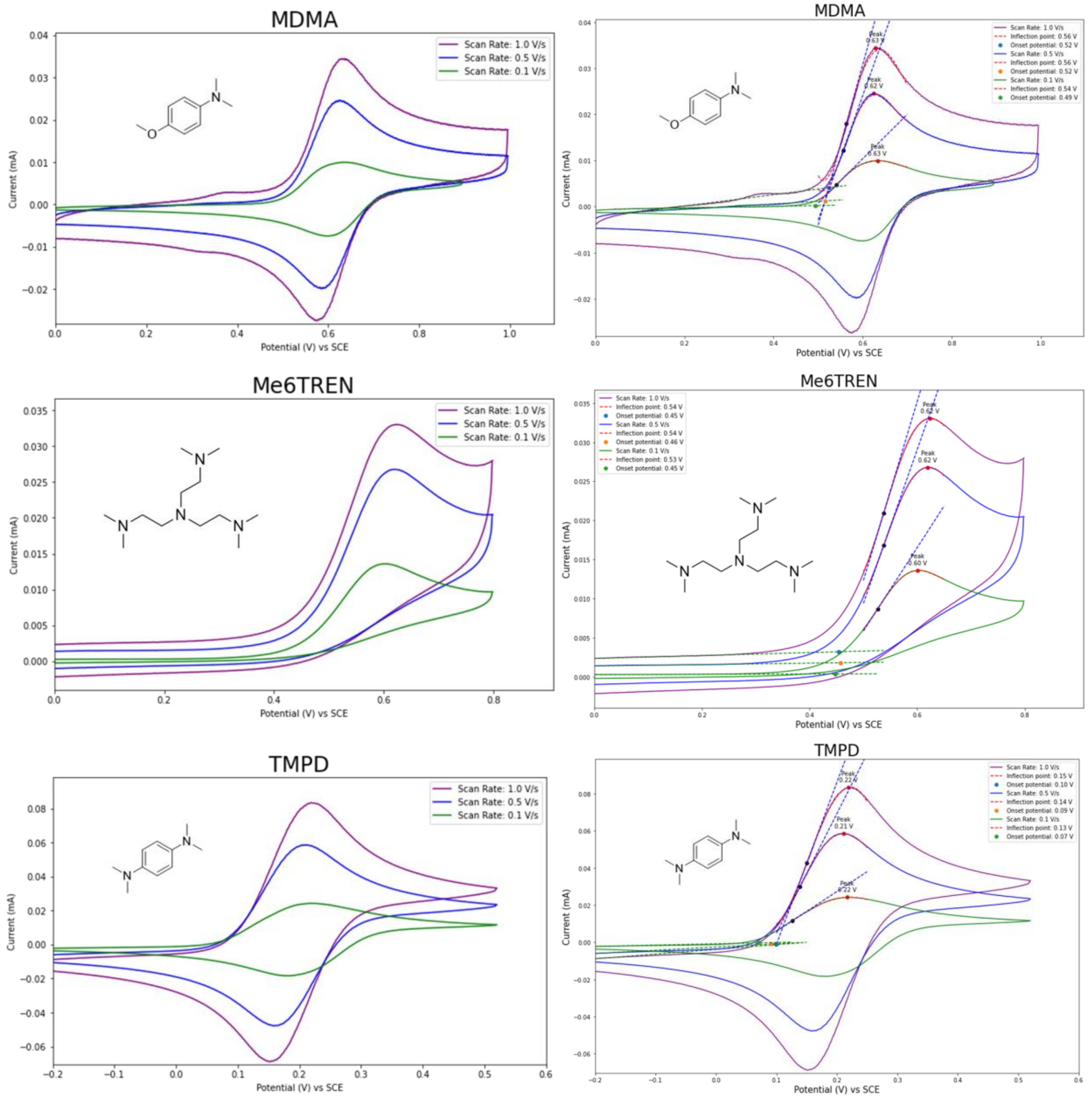

Figure S2. Cyclic voltammograms of the amines presented in Table 1 in MeCN at room temperature of amines at 1 mM analyte concentration with 0.1 M tetra-*n*-butylammonium hexafluorophosphate as the supporting electrolyte. The peak values were calculated with respect to SCE.

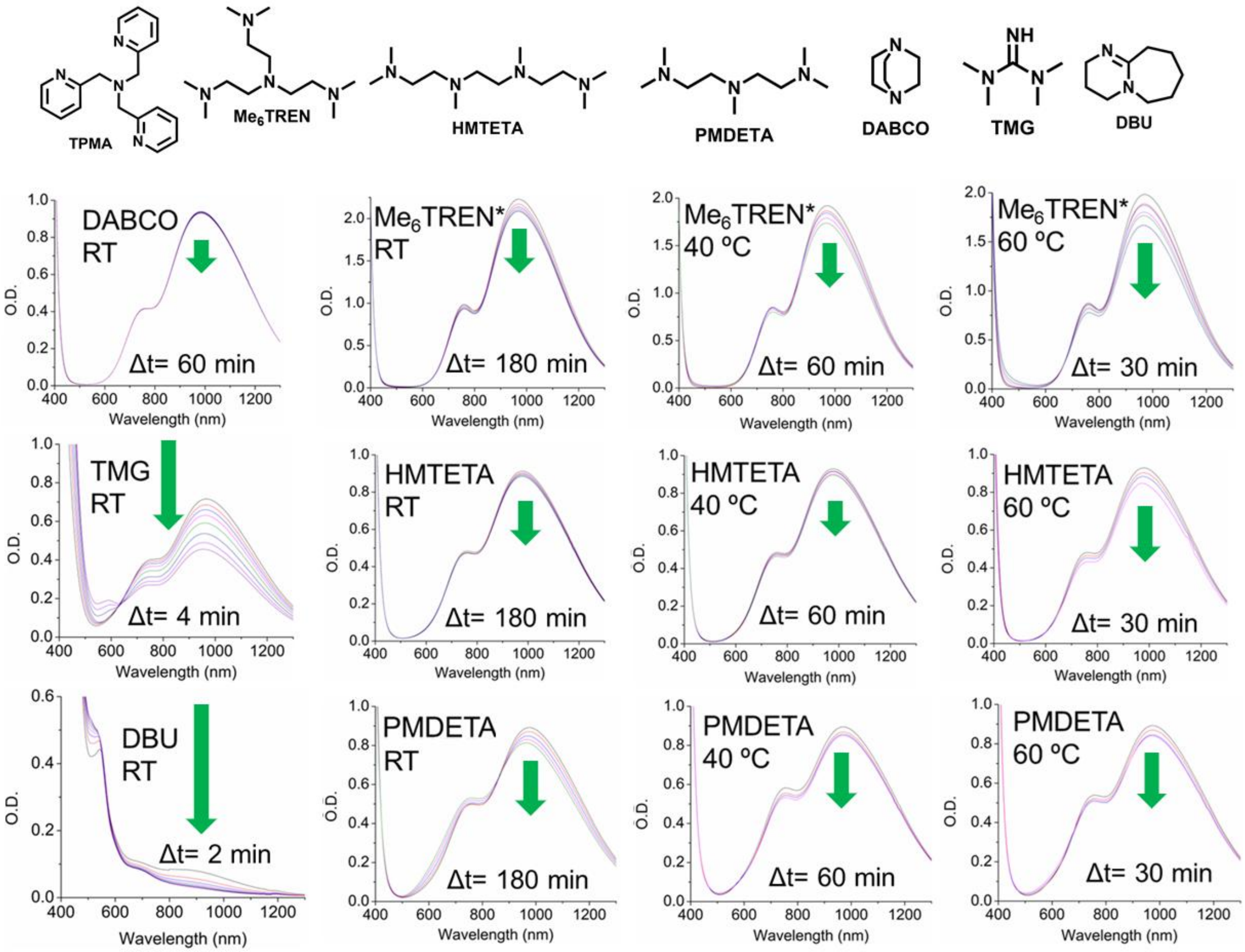

Figure S3. UV-Vis-NIR spectra recorded at different times for the reduction of $(Br\text{-}Cu^{II}/L)^{+}$ Br^{-} by excess amines as followed by the decrease in the absorption band at 960 nm for $(Br\text{-}Cu^{II}/TPMA)^{+}$ Br^{-} and 970 nm for $(Br\text{-}Cu^{II}/Me_6TREN)^{+}$ Br^{-}, ($[CuBr_2]_0$ = 4.55 mM, $[CuBr_2]_0/[TPMA]_0/[amine]_0$ = 1/1/5, no TPMA was used in the experiments with Me_6TREN: ($[CuBr_2]_0/[Me_6TREN]_0$ = 1/6) in MeCN.

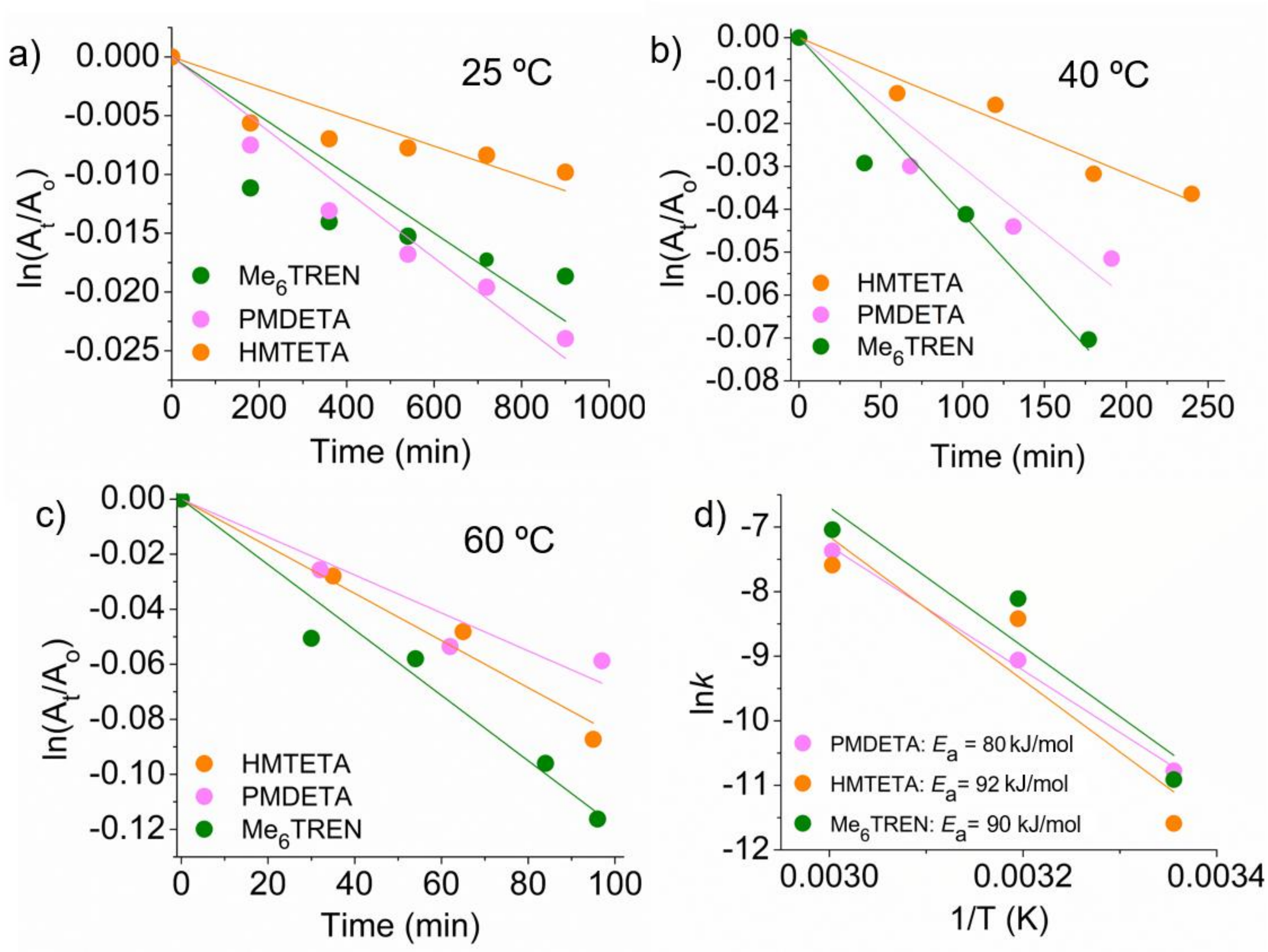

Figure S4. (a-c) Kinetics of the reduction of $(Br\text{-}Cu^{II}/L)^+$ Br^- at different temperatures by excess amines as followed by the decrease in the absorption band at 960 nm for $(Br\text{-}Cu^{II}/TPMA)^+$ Br^- and 970 nm for $(Br\text{-}Cu^{II}/Me_6TREN)^+$ Br^-, ($[CuBr_2]_0/[TPMA]_0/[HMTETA$ or $PMDETA]_0 = 1/1/5$), no TPMA was used in the experiments with Me_6TREN: ($[CuBr_2]_0/[Me_6TREN]_0 = 1/6$), $[CuBr_2]_0 = 4.55$ mM in DMSO. (d) Activation energies (E_a) of the reduction of $(Br\text{-}Cu^{II}/L)^+$ Br^- by excess amines.

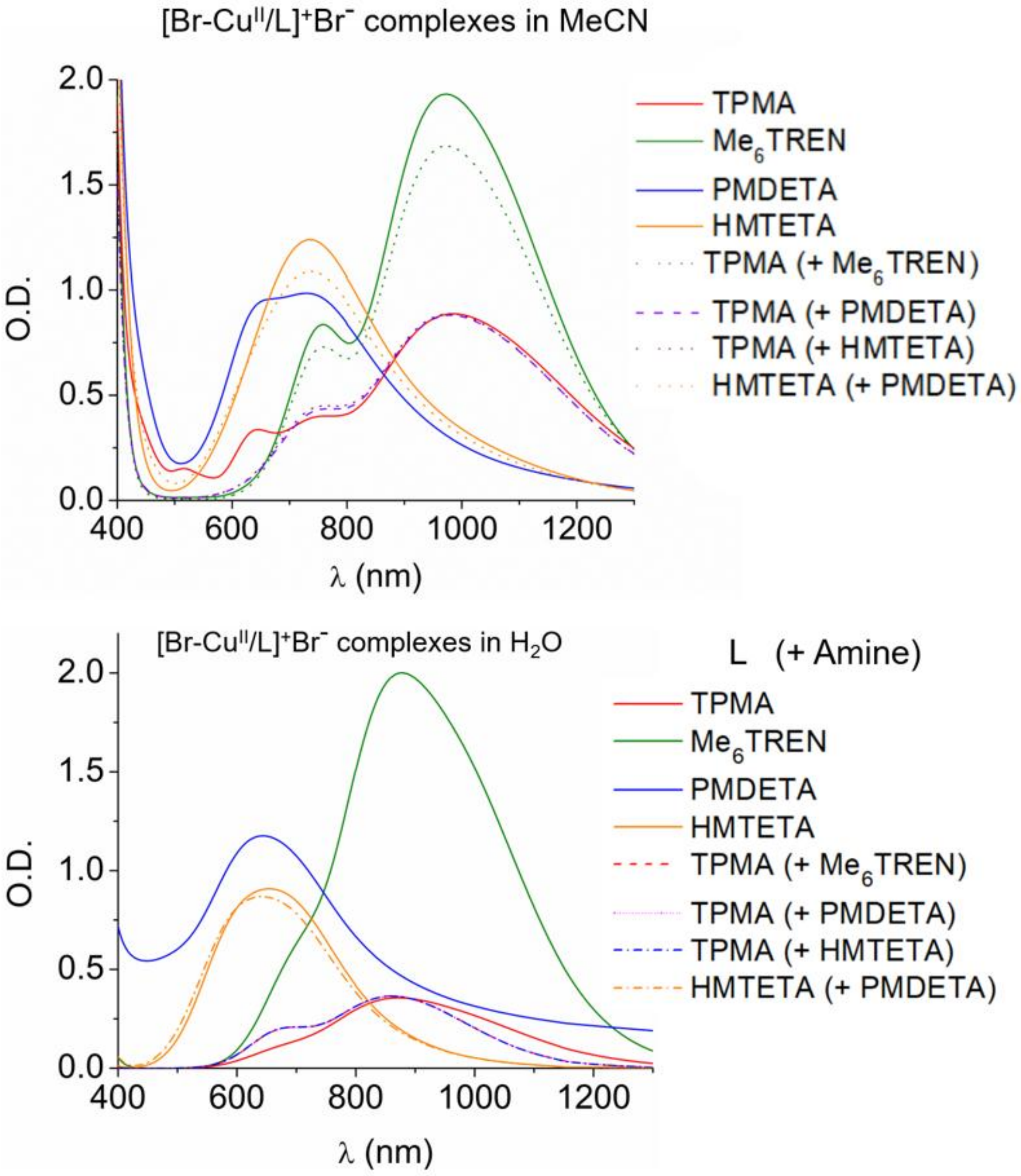

Figure S5. UV-Vis-NIR spectra of different [Br-Cu^{II}/L]$^+$Br$^-$ complexes in the absence/presence of an additional amine in MeCN (top) and water (bottom), ($[CuBr_2]_0$ = 4.55 mM, $[CuBr_2]_0/[Ligand]_0/[Amine]_0$ = 1/1/x, x = 0 or 1). The chelating strength of the ligands with $CuBr_2$ follows the order of Me$_6$TREN > TPMA > HMTETA > PMDETA in MeCN and TPMA > Me$_6$TREN > HMTETA > PMDETA in water. The UV-Vis-NIR was recorded immediately after the addition of new amines.

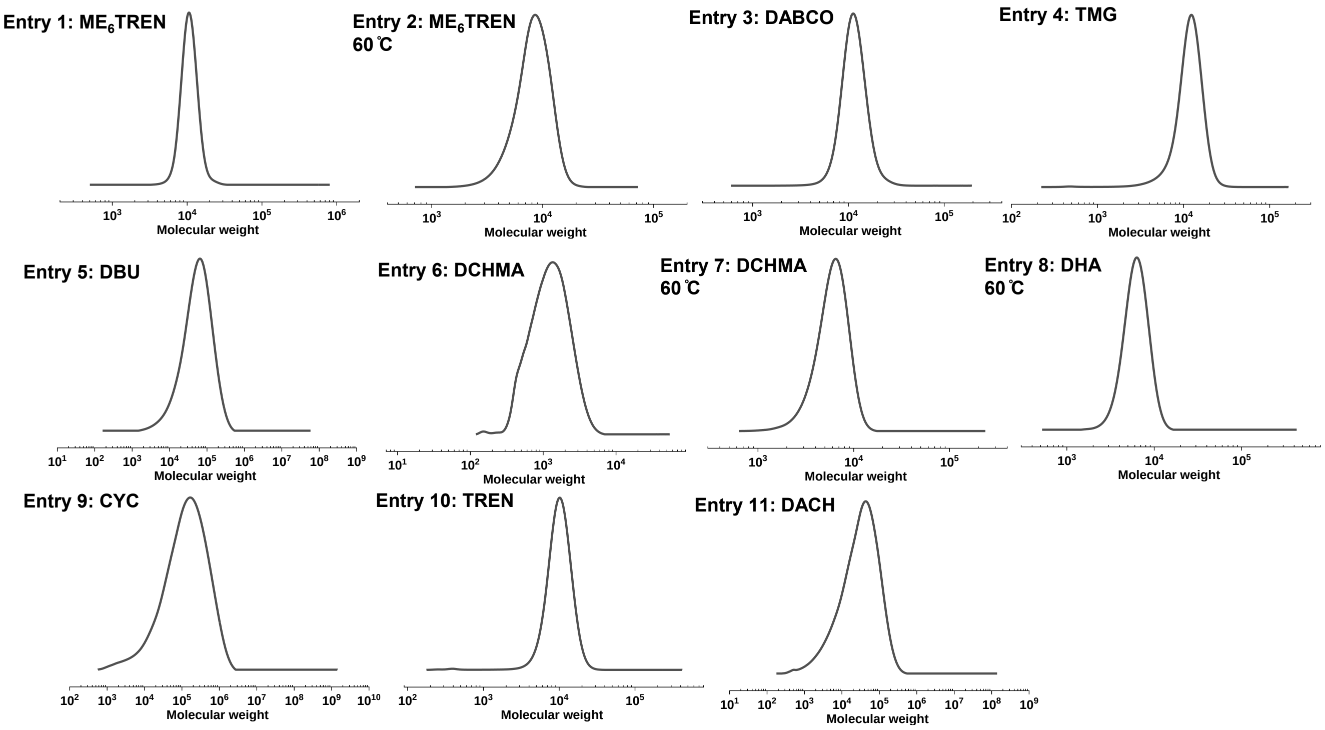

Figure S6. GPC traces for ATRP with $(Br\text{-}Cu^{II}/Me_6TREN)^+ Br^-$ or $(Br\text{-}Cu^{II}/TPMA)^+ Br^-$ and amines for polymerization results shown in Table 3.

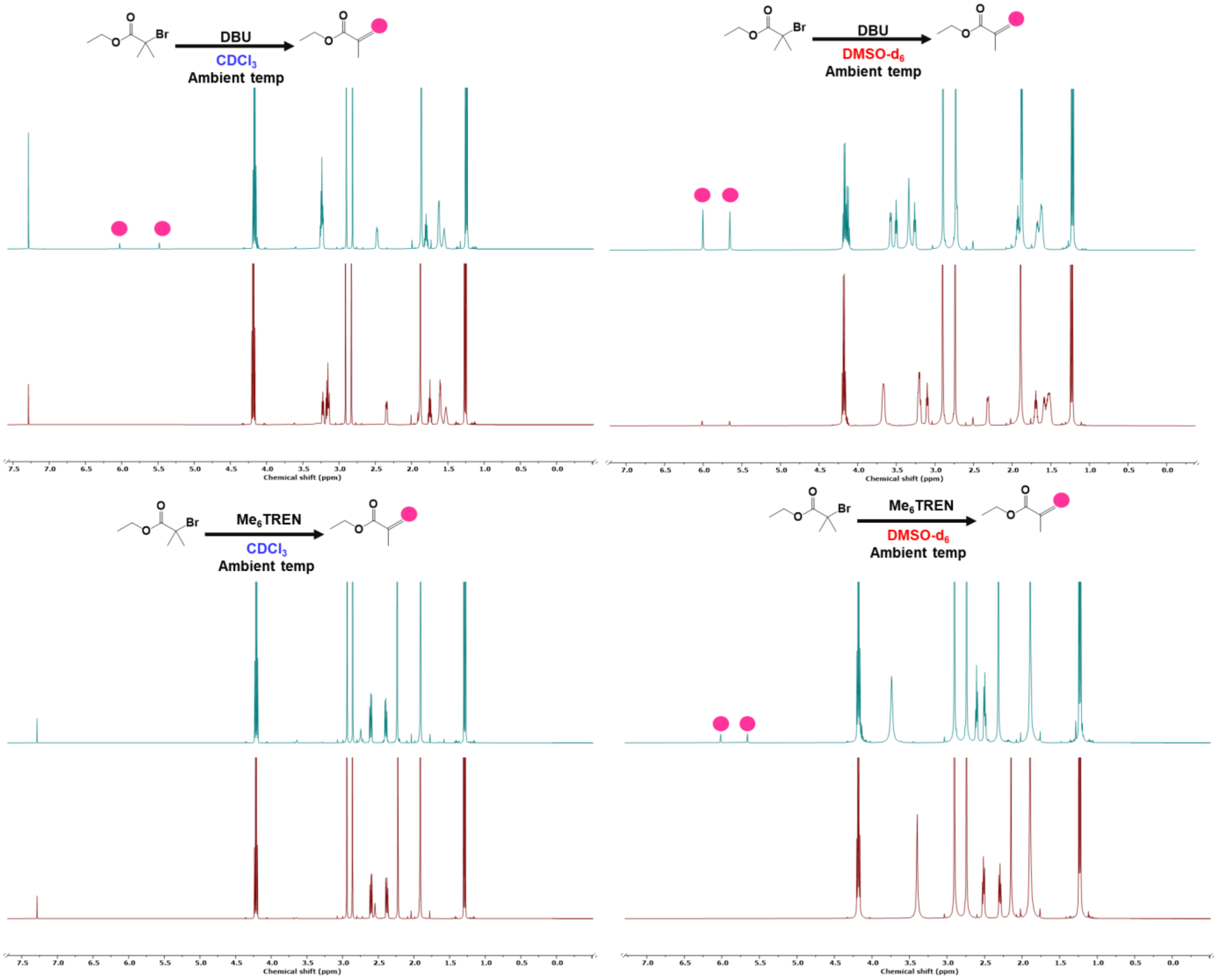

Figure S7. Elimination reaction of EBiB (224 mM) in the presence of DBU (89 mM, top) or Me_6TREN (89 mM, bottom) in $CDCl_3$ (left) or DMSO-d_6 (right) for 28 h. The elimination reaction was faster with DBU and in a more polar solvent (*i.e.*, DMSO-d_6).

Table S5. Results of ATRP of MA with $(Br\text{-}Cu^{II}/TPMA)^{+}\ Br^{-}$ and inactive amines.[a]

Entry	Amines	Time (h)	Temp (°C)	[b] Conv. (%)
1	TEOA	72	23	<5
2	TMED	72	23	<5
3	DMAE	72	23	<5
4	DMAEMA	72	23	<5
5	TMT	72	23	<5
6	PMDETA	72	23	<5
7	HMTETA	72	23	<5
8	TPMA	72	23	<5
9	PYR	72	23	<5
10	PIP	72	23	<5
11	TMPD	72	23	<5
12	MDMA	72	23	<5
13	BPY	72	23	<5
14	-	72	23	<5
15	TEOA	5.5	**60**	<5
16	TMED	5.5	**60**	<5
17	DMAE	5.5	**60**	<5
18	DMAEMA	5.5	**60**	<5
19	TMT	5.5	**60**	<5
20	PMDETA	5.5	**60**	<5
21	HMTETA	5.5	**60**	<5
22	TPMA	5.5	**60**	<5
23	PYR	5.5	**60**	<5
24	PIP	5.5	**60**	<5
25	TMPD	5.5	**60**	<5
26	MDMA	5.5	**60**	<5
27	-	5.5	**60**	<5

[a] Reaction conditions: $[MA]_0/[EBiB]_0/[CuBr_2]_0/[TPMA]_0/[amines]_0$ = 100/1/0.02/0.02/0.4 in DMSO, $[MA]_0$ = 5.8 M. [b] Calculated by ^{1}H-NMR.

Table S6. The results of ATRP with $(Br\text{-}Cu^{II}/TPMA)^+$ Br^- and ligands containing yellow impurities.[a]

Entry	Amines	Time (h)	Temp (°C)	Conv.[b] (%)	$M_{n,theo}$	[c]$M_{n,GPC}$	[c]$Đ$	I^* (%)
1	HMTETA	24	23	50	4500	4750	1.27	95
2	HMTETA	5.5	60	83	7300	10000	1.08	73
3	PMDETA	24	23	23	2200	2300	1.24	94
4	PMDETA	5.5	60	77	6800	7050	1.13	97
5	TPMA	24	23	<5	-	-	-	-
6	TPMA	5.5	60	72	6400	8300	1.08	77

[a] Reaction conditions: $[MA]_0/[EBiB]_0/[CuBr_2]_0/[TPMA]_0/[amines]_0$ = 100/1/0.02/0.02/0.4 in DMSO, $[MA]_0$ = 5.8 M. [b] Calculated by ^{1}H NMR. [c] Determined by GPC using PMMA standards.

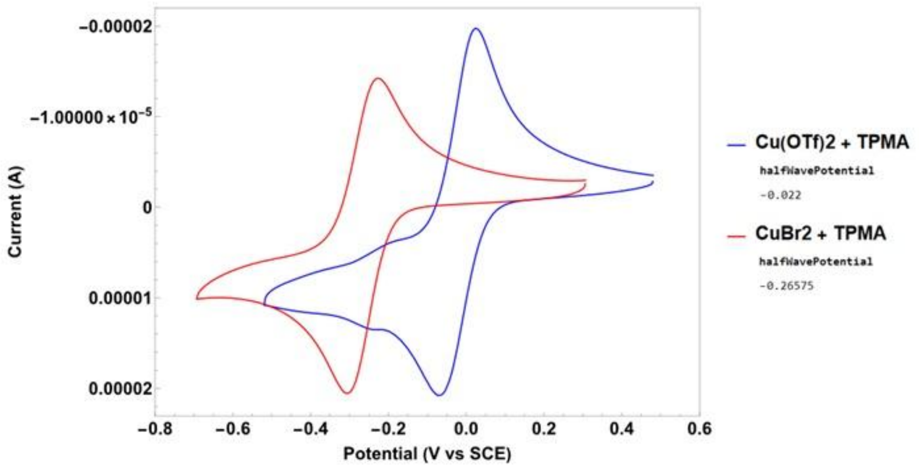

Figure S8. Cyclic voltammogram of the $(Br\text{-}Cu^{II}/TPMA)^{+}$ Br^{-} or $(Cu^{II}/TPMA)^{2+}$ $^{-}(OTf)_2$ in MeCN at room temperature. The oxidation peak values calculated with respect to the SCE.

Table S7. The results for ATRP with $(Cu^{II}/TPMA)^{2+}$ $^{-}(OTf)_2$ and inactive amines

Entry	Amines	Time (h)	Temp (°C)	[b] Conv. (%)
1	BPY	22	23	<5
2	PTH	22	23	<5
3	PG	22	23	<5
4	MDMA	22	23	<5
5	TMPD	22	23	<5

[a] Reaction conditions: $[MA]_0/[EBiB]_0/[Cu(OTf)_2]_0/[TPMA]_0/[amines]_0$ = 100/1/0.02/0.02/0.4 in DMSO, $[MA]_0$ = 5.8 M. [b] Calculated by ^{1}H NMR.

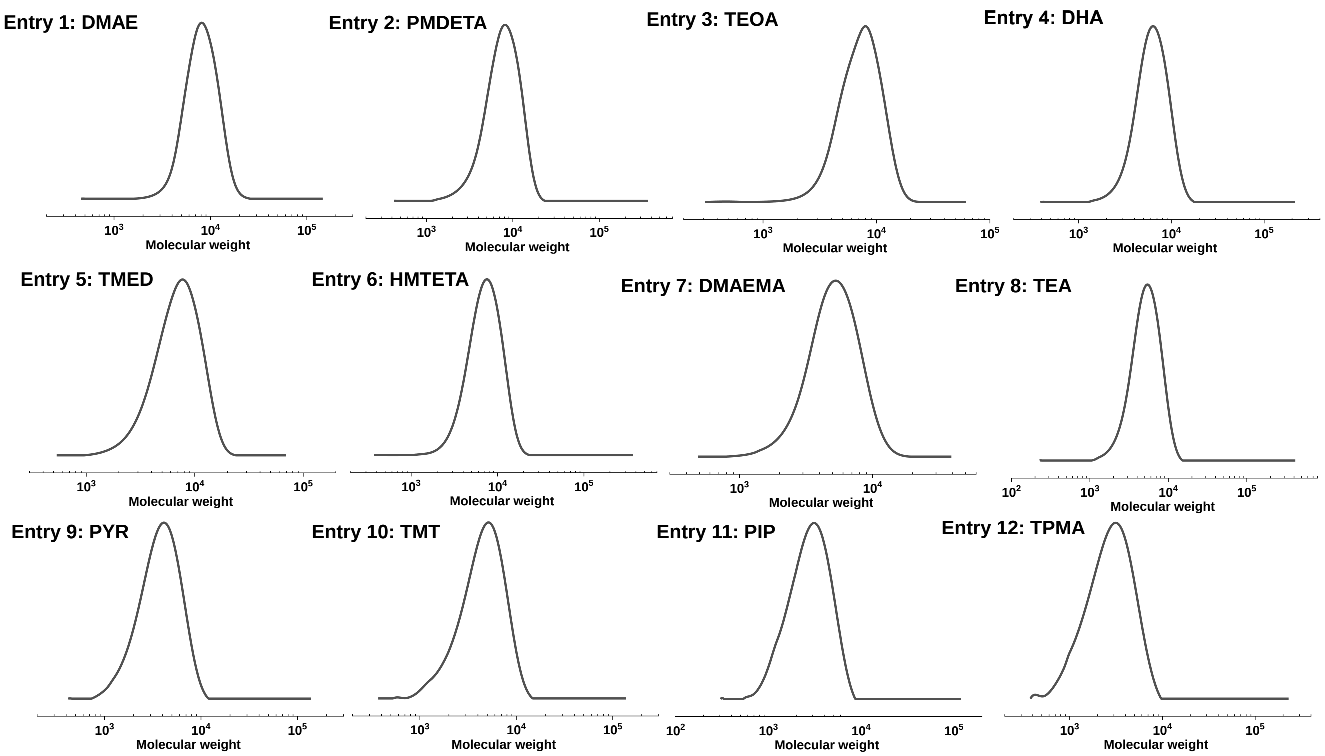

Figure S9. GPC traces for ATRP with $(Cu^{II}/TPMA)^{2+}$ $^{-}(OTf)_2$ and amines presented in Table 4.

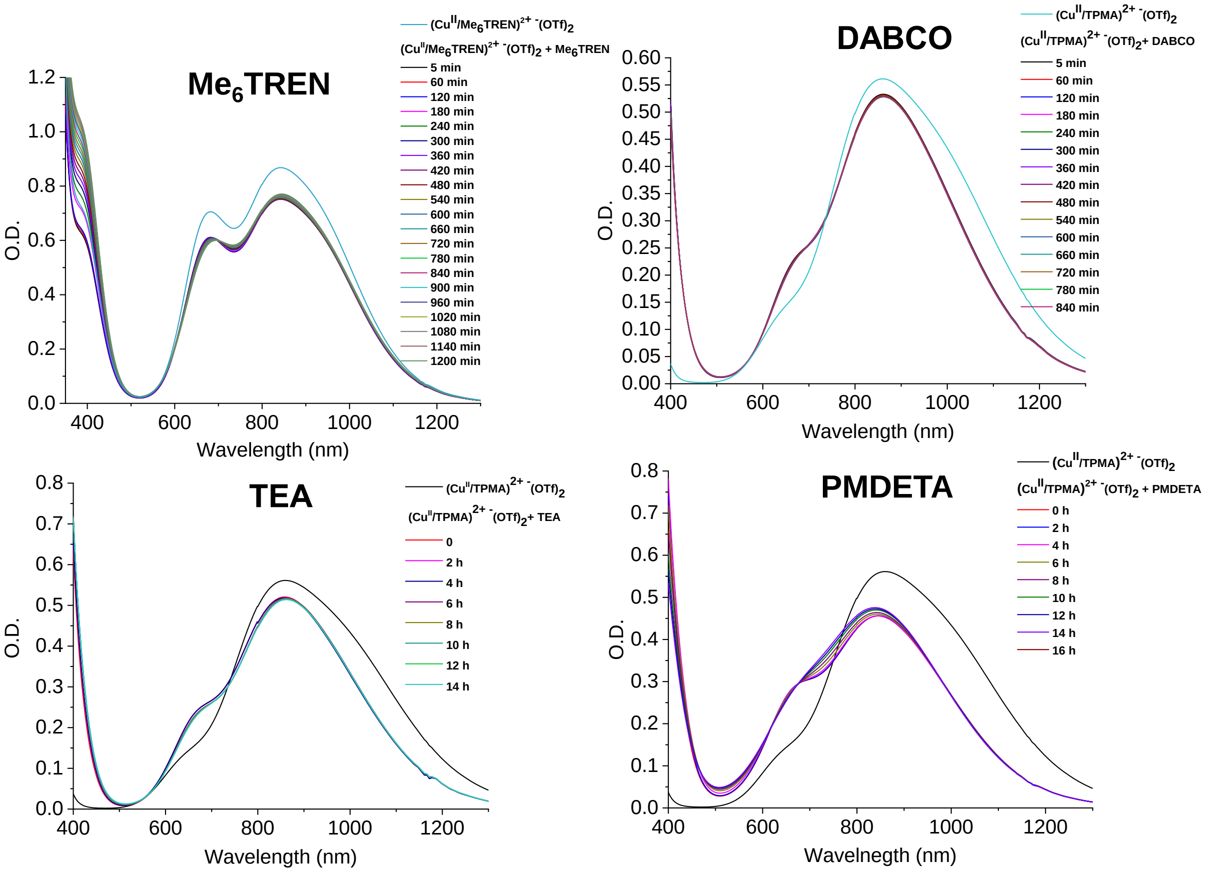

Figure S10. UV-Vis-NIR reduction of $(Cu^{II}/TPMA)^{2+}$ $^{-}(OTf)_2$ or $(Cu^{II}/Me_6TREN)^{2+}$ $^{-}(OTf)_2$ (2.7 mM) by Me_6TREN (top left), DABCO (top right), TEA (bottom left) and PMDETA (bottom right) in MeCN at room temperature under N_2 atmosphere.

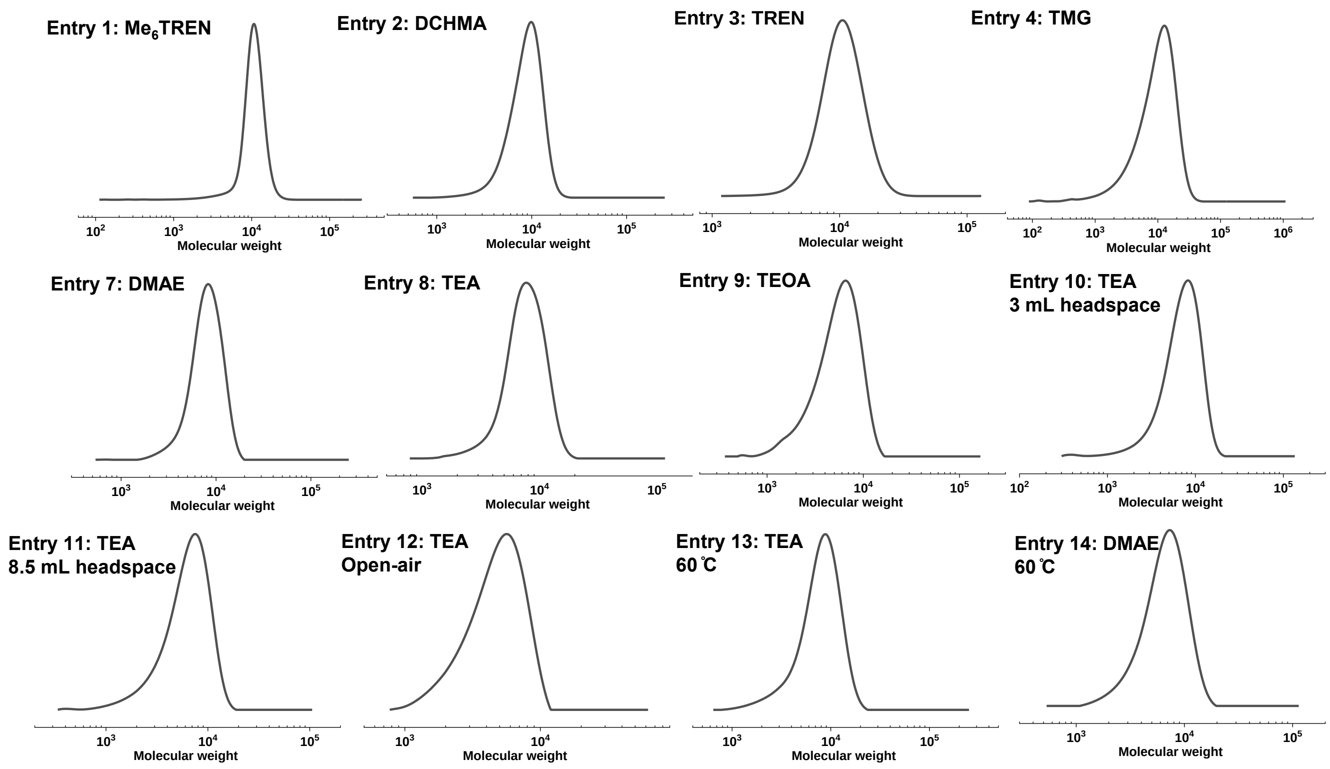

Figure S11. GPC traces for oxygen-tolerant ATRP with the $(Br\text{-}Cu^{II}/TPMA)^{+}\ Br^{-}$ or $(Cu^{II}/TPMA)^{2+}\ {}^{-}(OTf)_2$ and amines presented in Table 5.

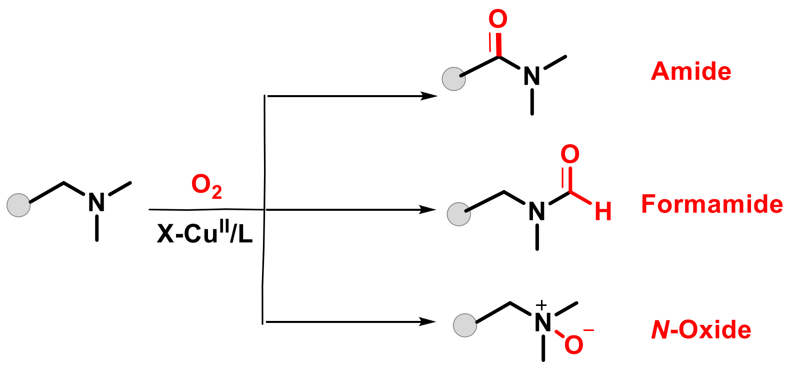

Figure S12. Possible reactions of amines with O_2 to form amide, formamide, or N-oxide.[2-5]

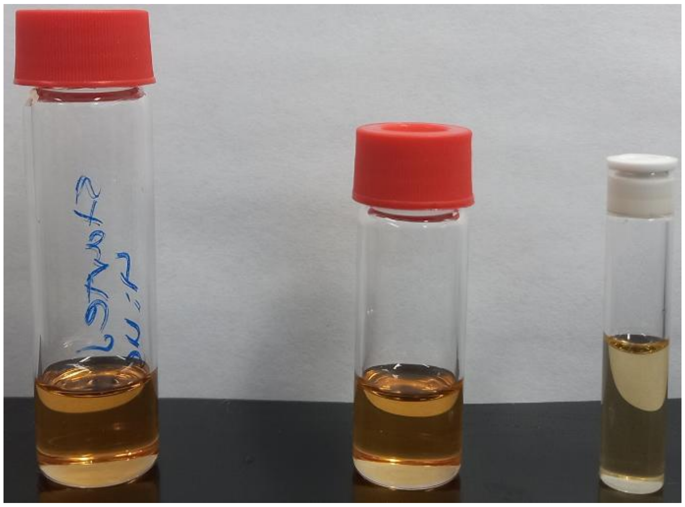

Figure S13. Digital images of oxygen-tolerant ATRP mixture with $(Cu^{II}/TPMA)^{2+}\ {}^{-}(OTf)_2$ and DMAE (entries 10-12, Table 5) showing the color change to yellow at the end of polymerization.

Table S9. Polymerization results of MA under varying targeted degrees of polymerization (DP).[a]

Targeted DP	Conv. (%)	$M_{n,theo}$	$M_{n,GPC}$	$Đ$	I^{*} (%)
50	97	4,350	4,100	1.15	106
100	97	8,550	9,250	1.08	92
200	97	17,100	18,500	1.06	92
400	83	27,420	35,150	1.07	78
800	82	56,600	49,300	1.09	115
1200	80	83,100	62,500	1.08	133

[a] Reaction conditions: $[MA]_0/[EBiB]_0/[CuBr_2]_0/[Me_6TREN]_0$ = 100/0.08-2/0.02/5 in MA/DMSO, v/v 1/1, under N_2 atmosphere after 18 h.

Table S10. Results of polymerization of MA with EBiB and amines via supplemental activation.[a]

Entry	Amines	Time (h)	Temp (°C)	[b] Conv. (%)
1	TREN	5.5	60	**24**
2	Me_6TREN	5.5	60	**47**
3	TPMA	5.5	60	**22**
4	PMDETA	5.5	60	**23**
5	TMG	5.5	60	<5
6	TMT	5.5	60	<5
7	TMED	5.5	60	<5
8	DABCO	5.5	60	<5
9	TEOA	5.5	60	<5
10	DHA	5.5	60	<5
11	PYR	5.5	60	<5
12	DCHMA	5.5	60	<5
13	DBU	5.5	60	<5
14	CYC	5.5	60	<5
15	PG	5.5	60	<5
16	MDMA	5.5	60	<5
17	TREN	72	**23**	<5
18	Me_6TREN	72	**23**	<5
19	TPMA	72	**23**	<5
20	PMDETA	72	**23**	<5
21	HMTETA	72	**23**	<5
22	DABCO	72	**23**	<5
23	TEOA	72	**23**	<5
24	CYC	72	**23**	<5
25	DHA	72	**23**	<5
26	DCHMA	72	**23**	<5

[a] Reaction conditions: $[MA]_0/[EBiB]_0/[amines]_0 = 100/1/0.4$ in DMSO, $[MA]_0 = 5.8$ M. [b] Calculated by ^{1}H NMR.

Supplemental activation of Cl-based initiator

A) Synthesis of the Cl-based initiator (ethyl 2-chloroisobutyrate, ECIB)

In a 300 mL round bottom flask equipped with a magnetic stirrer, ethanol (2.5 mL, 43 mmol) and triethyl amine (6 mL, 43 mmol) were dissolved in diethyl ether (150 mL) and the mixture was taken in an ice bath. To this mixture, 2-chloro-2-methylpropanoyl chloride (5 g, 35 mmol) in DCM (20 mL) was slowly added by an addition funnel. The mixture was left stirring overnight. The resulting solution was washed with aqueous solutions three times, dried over $MgSO_4$, and passed through a short column of silica to give the product as a pale-yellow liquid.

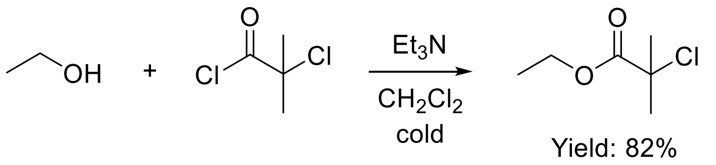

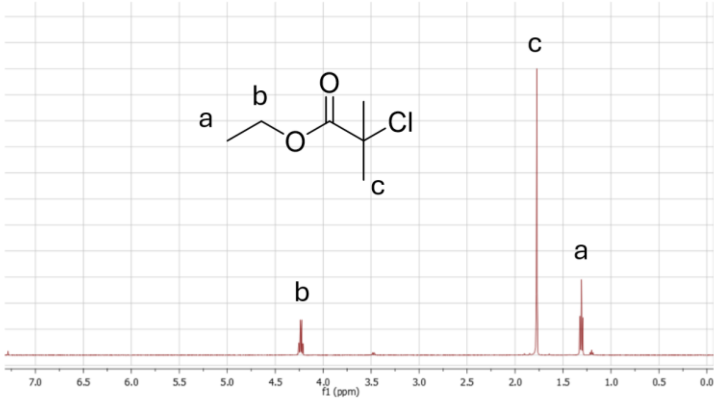

Figure S14. ^{1}H-NMR of ethyl 2-chloroisobutyrate (ECIB).

B) Polymerization of MA using ECIB and Me_6TREN (Supplemental Activation)

MA, ECIB, and Me_6TREN were mixed in a 5 mL volumetric flask in DMSO ($[MA]_0$ = 5.8 M, $[MA]_0/[ECIB]_0/[Me_6TREN]_0$ = 100/1/0.1). The mixture was transferred to a vial and purged with nitrogen for 10 mins. Then the vial was kept in an oil bath at 60 °C for 5.5 hours. After the reaction was quenched in an ice bath, an aliquot was for ^{1}H NMR analysis. No conversion was observed indicating that the alkyl halides could not be activated by excess amines, contrary to alkyl bromides.

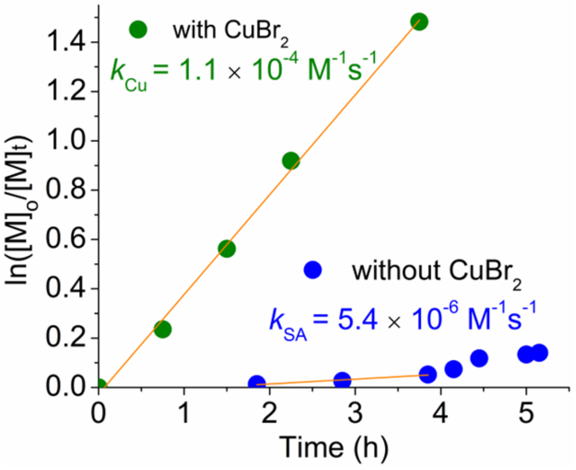

Figure S15. Comparison of the kinetics of MA polymerization in the presence (green dots) and absence (blue dots) of $CuBr_2$. Reaction conditions: $[MA]_0/[EBiB]_0/[CuBr_2]_0/[TPMA]_0/[amines]_0$ = 100/1/x/0.1, x = 0.02 or 0, in DMSO at 60 °C, $[MA]_0$ = 5.8 M. The slopes give k_{Cu} = 1.1 × 10^{-4} $M^{-1}s^{-1}$ and k_{SA} = 5.4 × 10^{-6} $M^{-1}s^{-1}$.

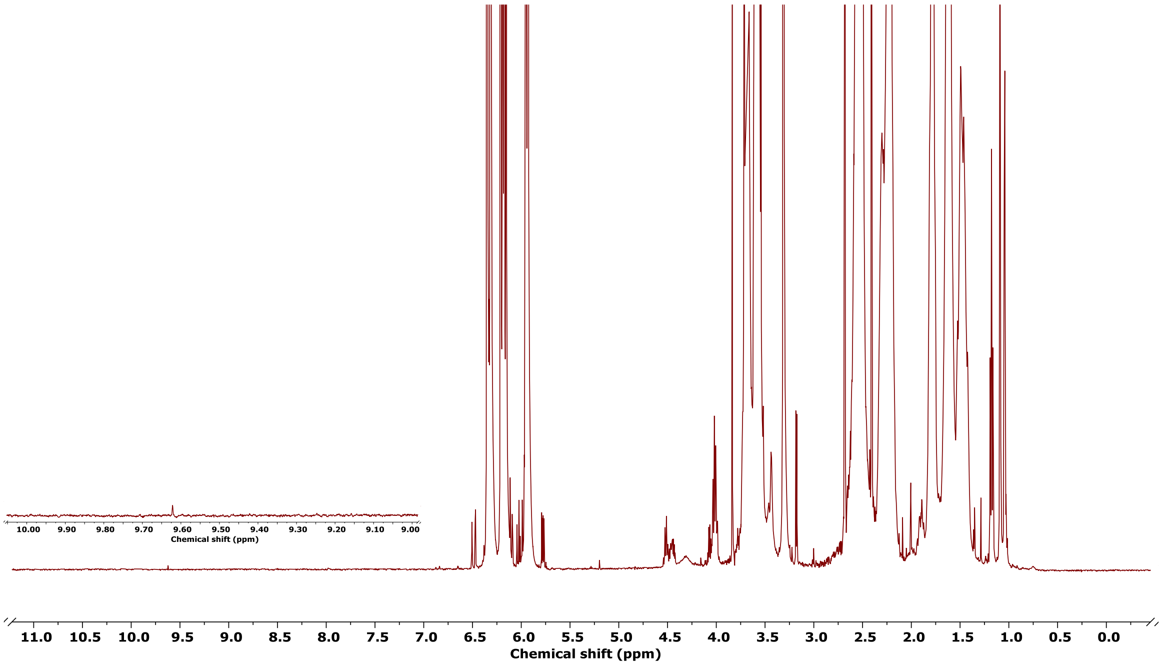

Figure S16. ^{1}H-NMR of polymerization mixture in the presence of amines showing formation a new peak at 9.6 ppm.

Predici simulations of ATRP in the presence of amines as reducing agents in the dark

A regenerative ATRP system was modeled by Predici v11.0 (moment mode, 0.0001 accuracy, simulation time = 86400 s) according to the proposed reduction mechanism by amines.[6] The conditions represent that of Table 2, Entry 1 in the main text. Generally, polymerization rate constants were set as seen in literature at room termperature. The following assumptions were employed

1. At room temperature, radical cation amine ($R_3N^{\bullet+}X^-$) generated as a product of reduction cannot appreciably initiate new chains (as per Figure 3b, main text) and any further decomposition reactions of them are deemed negligible concerning ATRP.
2. Conventional radical termination of poly(acrylates) was assumed to proceed primarily through radical combination.
3. The formation of organometallics from polymeric radicals and Cu^I/L^+ activators was deemed negligible due to the slow reduction and low formation of Cu^I/L^+.
4. The loading of amine was scaled by the four amine moieties in Me_6TREN, meaning that each amine in Me_6TREN is equivalent and can act as a reducing agent. Likewise with slow reduction, amines do not appreciably decompose, e.g. $[R_3N] \approx [R_3N]_0$.

With these assumptions, macromolecular simulations provided polymerization conversion, dispersity, as well as tracking of species and associated reaction rates based on Table S11.

Table S11. Predici simulation model for ATRP of methyl acrylate in the presence of amines in the dark.

Reactions	Reactant 1	(Reactant 2)	Product 1	(Product 2)	*k*	*value*	Reference
Radical Addition	$R^\bullet$	M	$P_1^\bullet$		k_{add}	1.4×10^3	7, 8
Propagation	$P_n^\bullet$	M	$P_{n+1}^\bullet$		k_p	1.5×10^4	9
ATRP Activation	R-Br	$[Cu^I/L]^+$	$R^\bullet$	$[Br\text{-}Cu^{II}/L]^+$	$k_{a,R\text{-}X}$	3.1×10^4	9
	P_n-Br	$[Cu^I/L]^+$	$P_n^\bullet$	$[Br\text{-}Cu^{II}/L]^+$	$k_{a,P\text{-}X}$	3.8×10^3	9
ATRP Deactivation	$R^\bullet$	$[Br\text{-}Cu^{II}/L]^+$	R-Br	$[Cu^I/L]^+$	$k_{d,R\text{-}X}$	8.75×10^6	9
	$P_n^\bullet$	$[Br\text{-}Cu^{II}/L]^+$	P_n-Br	$[Cu^I/L]^+$	$k_{d,P\text{-}X}$	5.6×10^7	9
Conventional Radical Termination	$P_n^\bullet$	$P_s^\bullet$	P_n-P_s		k_t	2.4×10^8	9
Reduction	$[Br\text{-}Cu^{II}/L]^+$	R_3N	$[Cu^I/L]^+$	$R_3N^{\bullet+}Br^-$	$k_{red(exp)}$	1.8×10^{-5}	*This Work*

Reaction conditions: $[MA]_0/[EBiB]_0/[Br\text{-}Cu^{II}/L]^+_0/[R_3N]_0$= 100/1/0.02/0.4 in DMSO, where L = Me_6TREN and $[MA]_0$ = 5.8 M. Ligated equivalents of Me_6TREN were loaded explicitly in $[Br\text{-}Cu/Me_6TREN]^+$ complex and differed from free Me_6TREN which was simplified as R_3N moieties. *This work* refers to Main Text, Table 2 Entry 1. [a] EBiB scaled from analogous methyl 2-bromopropionate initiator modeling PMA-Br chains ends based on literature.[10]

The monomer conversion, concentration of species, were output with respect to time and reaction rates calculated on the basis of the rate constant in Table S11 times the concentration of reactants.

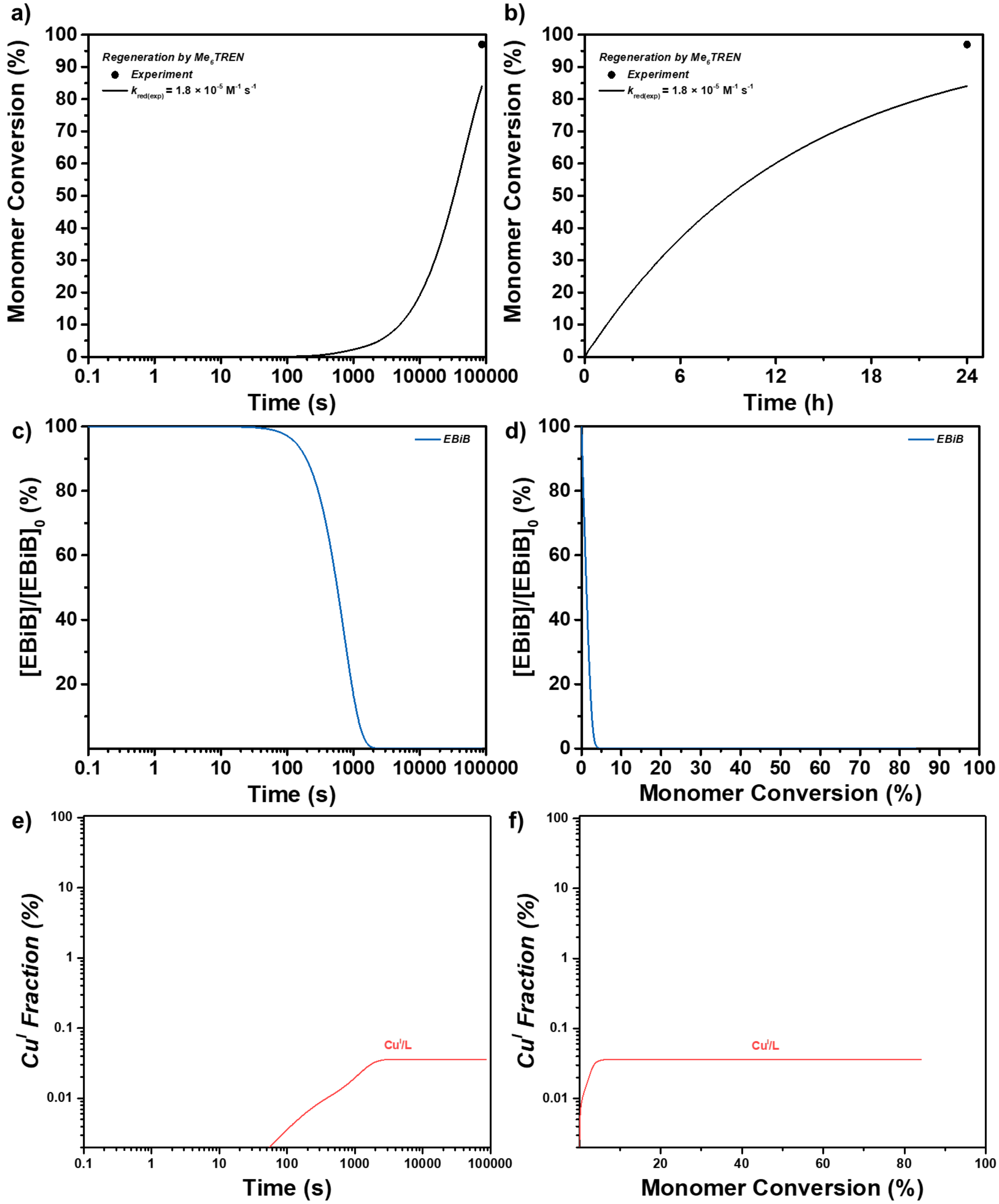

Figure S17. Predici simulation plots of a) monomer conversion vs (logarithmic) time, b) monomer conversion vs (linear) time, c) remaining fraction (%) of EBiB vs (logarithmic) time, d) remaining fraction (%) of EBiB vs monomer conversion (%), e) Cu^I/L species as (%) fraction of loaded Cu vs (logarithmic) time, and f) Cu^I/L species as (%) fraction of loaded Cu vs monomer conversion (%). Simulation conditions: $[MA]_0/[EBiB]_0/[Br\text{-}Cu^{II}/Me_6TREN]^+_0/[R_3N]_0 = 100/1/0.02/0.4$, where R_3N are amine moieties of free Me_6TREN.

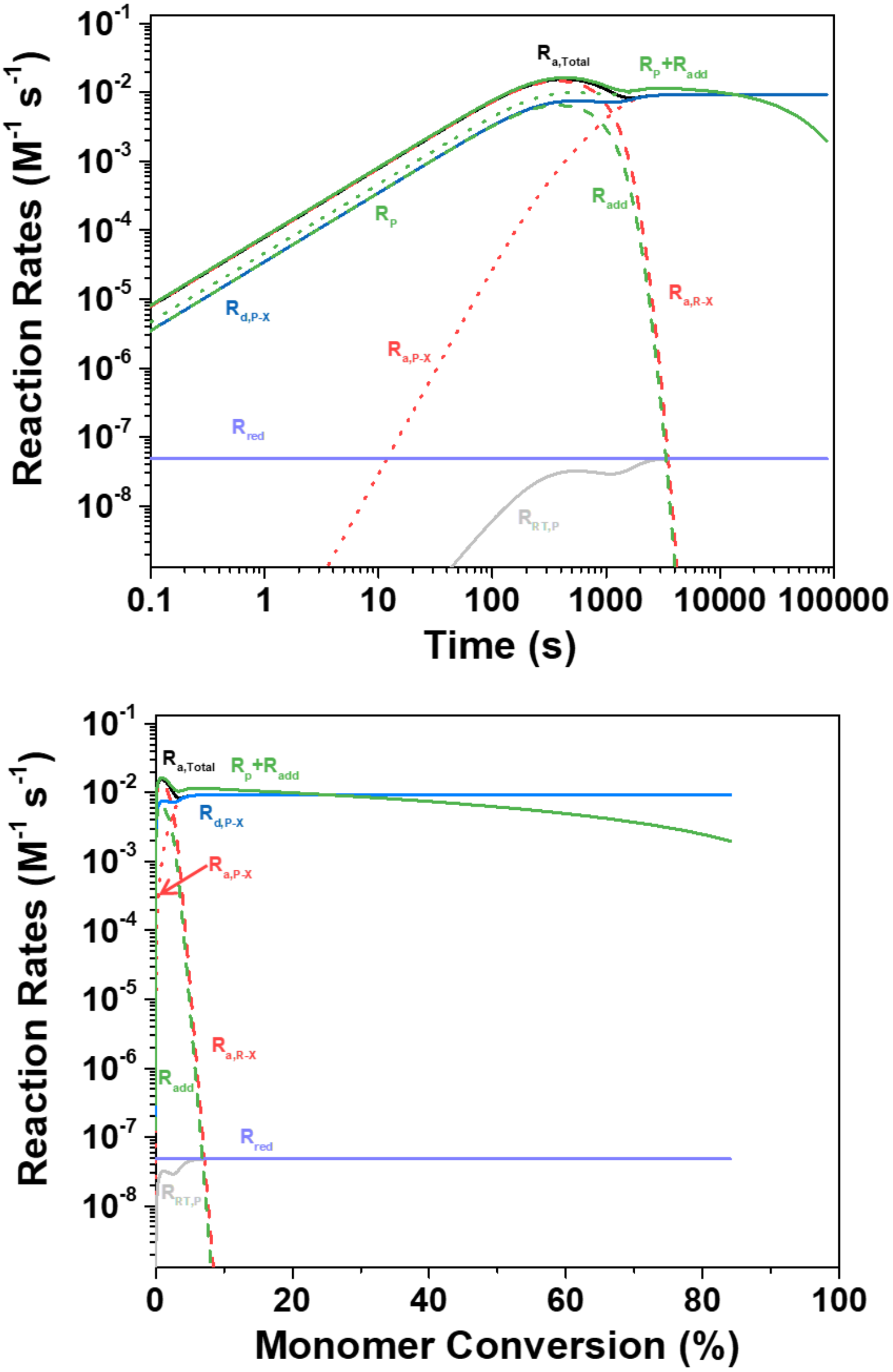

Figure S18. Reaction rates calculated as products of rate constants and concentrations of reactants vs (top) logarithmic time or (bottom) monomer conversion (%). Simulation conditions: $[MA]_0/[EBiB]_0/[Br\text{-}Cu^{II}/Me_6TREN]^+_0/[R_3N]_0$= 100/1/0.02/0.4, where R_3N are amine moieties of free Me_6TREN. $R_{a,P\text{-}X}$ – activation of polymeric alkyl halide ($k_{a,P\text{-}X}$, dotted red); $R_{a,R\text{-}X}$ – activation of alkyl halide initiator (dashed

red); $R_{a,total}$ – total activation (black); $R_{d,P\text{-}X}$ – deactivation of polymeric radicals (blue); R_{add} – addition (dashed green); R_P – polymerization (green, combined with R_{add}); R_{red} – reduction (purple); $R_{RT,P}$ – conventional radical termination (polymeric radicals, gray).

The simulation showcases a typical regenerative ATRP system. The system reaches a steady-state in which the rates of activation and deactivation become equal. Afterwards, the rate of polymerization (R_P) decreases with time. Overall, a high conversion of polymerization was reached alongside a low final dispersity of 1.03 at 24 h.

The simulation shows the steady-state relationship of reduction and termination whereby they equal one another. Termination is proportional to the square of radical concentration; hence, any faster reduction rate will yield more radicals and faster polymerization. This is a well-established steady-state relationship in regenerative ATRP and is also observed in a steady-state model of similar conditions in literature.[9] The amount of Cu in the forms of Cu^I/L activator and $Br\text{-}Cu^{II}/Me_6TREN^+$ was output as a fraction of the loaded Cu to confirm the small amount of reduction overall. Figure S17 shows that the fraction of Cu in the Cu^I/L activator form is <0.1% maintained consistently through the simulation at the steady state. Hence, an overall low reduction turnover of Cu at ppm levels by amine can still drive polymerization to completion.

The corresponding pre-steady state between EBiB and PMA-Br chain ends was modelled due to their difference in activities. The time where EBiB remains is approximately the first ~1000 seconds. This corresponds to <5% of monomer conversion by which all EBiB is activated and consumed. The inflection point in the radical concetration (inferred from termination/propagation), activation/deactivation, and propagation marks the conversion of R-X into P-X, before reaching the final ATRP steady state.

References

(1) Jazani, A. M.; Schild, D. J.; Sobieski, J.; Hu, X.; Matyjaszewski, K. Visible Light-ATRP Driven by Tris(2-Pyridylmethyl)Amine (TPMA) Impurities in the Open Air. *Macromolecular Rapid Communications* **2023**, *44* (16), 2200855.
(2) Zhang, Y.; Riemer, D.; Schilling, W.; Kollmann, J.; Das, S. Visible-Light-Mediated Efficient Metal-Free Catalyst for α-Oxygenation of Tertiary Amines to Amides. *ACS Catalysis* **2018**, *8* (7), 6659-6664.
(3) Nakai, S.; Yatabe, T.; Suzuki, K.; Sasano, Y.; Iwabuchi, Y.; Hasegawa, J.-y.; Mizuno, N.; Yamaguchi, K. Methyl-Selective α-Oxygenation of Tertiary Amines to Formamides by Employing Copper/Moderately Hindered Nitroxyl Radical (DMN-AZADO or 1-Me-AZADO). *Angewandte Chemie International Edition* **2019**, *58* (46), 16651-16659.
(4) Lalevée, J.; Graff, B.; Allonas, X.; Fouassier, J. P. Aminoalkyl Radicals: Direct Observation and Reactivity toward Oxygen, 2,2,6,6-Tetramethylpiperidine-N-oxyl, and Methyl Acrylate. *The Journal of Physical Chemistry A* **2007**, *111* (30), 6991-6998.
(5) Mandigma, M. J. P.; Žurauskas, J.; MacGregor, C. I.; Edwards, L. J.; Shahin, A.; d'Heureuse, L.; Yip, P.; Birch, D. J. S.; Gruber, T.; Heilmann, J.; et al. An organophotocatalytic late-stage N–CH3 oxidation of trialkylamines to N-formamides with O2 in continuous flow. *Chemical Science* **2022**, *13* (7), 1912-1924, 10.1039/D1SC05840A.
(6) Wulkow, M. Computer Aided Modeling of Polymer Reaction Engineering—The Status of Predici, I-Simulation. *Macromolecular Reaction Engineering* **2008**, *2* (6), 461-494.
(7) Krys, P.; Fantin, M.; Mendonça, P. V.; Abreu, C. M. R.; Guliashvili, T.; Rosa, J.; Santos, L. O.; Serra, A. C.; Matyjaszewski, K.; Coelho, J. F. J. Mechanism of supplemental activator and reducing agent atom transfer radical polymerization mediated by inorganic sulfites: experimental measurements and kinetic simulations. *Polymer Chemistry* **2017**, *8* (42), 6506-6519, 10.1039/C7PY01319A.

(8) Martinez, M. R.; Sobieski, J.; Lorandi, F.; Fantin, M.; Dadashi-Silab, S.; Xie, G.; Olszewski, M.; Pan, X.; Ribelli, T. G.; Matyjaszewski, K. Understanding the Relationship between Catalytic Activity and Termination in photoATRP: Synthesis of Linear and Bottlebrush Polyacrylates. *Macromolecules* **2020**, *53* (1), 59-67.
(9) Whitfield, R.; Parkatzidis, K.; Bradford, K. G. E.; Truong, N. P.; Konkolewicz, D.; Anastasaki, A. Low ppm CuBr-Triggered Atom Transfer Radical Polymerization under Mild Conditions. *Macromolecules* **2021**, *54* (7), 3075-3083.
(10) Tang, W.; Kwak, Y.; Braunecker, W.; Tsarevsky, N. V.; Coote, M. L.; Matyjaszewski, K. Understanding Atom Transfer Radical Polymerization: Effect of Ligand and Initiator Structures on the Equilibrium Constants. *Journal of the American Chemical Society* **2008**, *130* (32), 10702-10713.
